# Tobacco-related lung cancer burden in the Western Pacific Region from 1990 to 2021: An age-period-cohort analysis from the Global Burden of Disease Study

**DOI:** 10.18332/tid/201970

**Published:** 2025-03-13

**Authors:** Rui Wang, Zhiqiang Zhang, Xiaoxi Shan, Jiayang Dong, Xinyue Yang, Jing Zhang, Jie Cao

**Affiliations:** 1 Department of Respiratory and Critical Care Medicine, Tianjin Medical University General Hospital, Tianjin, China; 2 Graduate School of Tianjin Medical University, Tianjin Medical University, Tianjin, China

**Keywords:** Western Pacific Region, tobacco, lung cancer, ageperiod-cohort analysis

## Abstract

**INTRODUCTION:**

Lung cancer is a significant health issue in the Western Pacific region, where tobacco use is highly prevalent. This study examines the trends in tobacco-related lung cancer burden from 1990 to 2021, offering insights into the evolution of this critical public health challenge.

**METHODS:**

This study uses data from the Global Burden of Disease (GBD) 2021 study to analyze lung cancer rates. It employs an age-period-cohort analysis to explore patterns in mortality and disability-adjusted life-years (DALYs) attributed to tobacco-related lung cancer. The study also uses joinpoint regression analysis to pinpoint changes over different periods.

**RESULTS:**

Our analyses revealed a substantial rise in both the number of deaths and DALYs due to tobacco-related lung cancer in the Western Pacific region from 1990 to 2021. Total deaths increased by 163.42% to 644.5 thousand (95% UI: 517.9–793.8) in 2021, which is 2.6 times the global growth rate of 63.25%. While global ASMR decreased by 25.46%, the Western Pacific Region showed minimal change with a slight increase (AAPC=0.08). SDI analysis revealed an inverse relationship with burden – for example, Singapore's ASMR decreased from 20.4 to 7.0 per 100000 population (high SDI) while China's increased from 23.8 to 25.8 (middle SDI). Age-period-cohort analysis showed the net drift of lung cancer mortality was -0.16% per year. The fastest increase in ASMR occurred between 1998–2004 (APC=1.53%), followed by the steepest decline during 2004–2007 (APC= -1.49%).

**CONCLUSIONS:**

The Western Pacific region continues to face a high burden of tobacco-related lung cancer.

## INTRODUCTION

Lung cancer (including trachea, bronchus, and lung cancer) is the leading cause of cancer-related deaths and the second most common cancer globally^1^. There are approximately 2 million new cases of lung cancer and 18.01 million lung cancer deaths each year^2,3^. In the global cancer statistics for 2020, lung cancer accounted for 18% of cancer-related deaths^4^. Smoking, air pollution, genetics, and radiation are risk factors for lung cancer^5,6^.

Cigarettes are a major risk factor for lung cancer and can produce various carcinogens (including tobacco-specific nitrosamines, polycyclic aromatic hydrocarbons, and volatile organic compounds)^7^. Smoking is closely related to the occurrence, progression, metastasis, drug resistance, and poor prognosis of lung cancer^8,9^. Long-term smokers are 20 times more likely to develop lung cancer compared to non-smokers^10^, and 85% of lung cancer cases are associated with smoking^11,12^. There are over 1 billion smokers worldwide^13,14^. In 2019, the global consumption of tobacco was 7.41 trillion (range: 7.11–7.74) cigarette-equivalents^15,16^. Although global tobacco control measures have been effective in reducing tobacco use worldwide, there are differences in the levels of implementation across regions, and the global tobacco epidemic remains at a high level.

In the 2019 Global Burden of Disease (GBD) data on global cancer, lung cancer accounted for 18.3% of DALYs^17^. In 2019, 80.3% of lung cancer deaths were attributable to risk factor exposure, with the top three risk factors being smoking, environmental particulate matter pollution, and occupational asbestos exposure^18^. In the six global regions defined by the World Health Organization (WHO), the Western Pacific region includes countries that account for one-quarter of the world’s population^19^.

In recent years, with ongoing social and economic development, the average life expectancy in the Western Pacific Region has continued to increase, exacerbating population aging. According to data from the WHO, the rate of growth in the proportion of elderly people in the Western Pacific region exceeds that of other age groups^20^. The ‘Western Pacific Regional Action Plan for the Prevention and Control of Noncommunicable Diseases 2014-2020’ was established in 2013, aiming to reduce deaths in the Western Pacific Region caused by noncommunicable diseases^21^. The rapid increase in the elderly population has exacerbated the cancer burden in the Western Pacific region. This study comprehensively assesses the tobacco-related lung cancer burden in the Western Pacific Region by analyzing data from the Global Burden of Disease Study 2021 (1990–2021), aiming to provide evidence-based insights for tobacco control policies and interventions to reduce the substantial lung cancer burden in this region.

## METHODS

### Data source

Our analysis is a secondary analysis based on the GBD 2021 database (http://ghdx.healthdata.org/gbd-results-tool), which includes global incidence, prevalence, years lived with disability (YLD), disability-adjusted life years (DALYs), and healthy life expectancy (HALE) for 371 diseases and injuries in 204 regions and countries, and 811 localities^22^. By calculating the detailed data of tobacco-related lung cancer burden provided by the database, we determined the trends in deaths and DALYs due to lung cancer caused by tobacco use. The methods used to generate these values are similar to those used in the 2019 GBD^23^. To ensure consistency, tobacco is categorized into three types: tobacco use, smoking, and secondhand smoke. The study population is divided into five age groups: 25–49, 50–59, 60–69, 70–79, and ≥80 years, as the number of cases was too small in populations <50 years and sparse in those over 80 years. The Sociodemographic Index (SDI) is divided into five categories based on the score (0–1): high, high-middle, middle, low-middle, and low. GBD is performed in compliance with Guidelines for Accurate and Transparent Health Estimates Reporting (GATHER)^24^.

### Definition of tobacco exposure

Consistent with the assessment methods used for the 2019 GBD risk factors, a comprehensive evaluation was conducted on risk factor exposure levels, relative risks, and disease burden^14^. We classified the tobacco-exposed population into two groups: smokers and secondhand smoke exposure individuals. Due to differences in information collection, there may be overlap between these two groups. Smoking is defined as the current or past occasional or daily use of tobacco products, including pipes, cigarettes, cigars, waterpipes, bidis, and other locally used tobacco products^15^. Secondhand smoke exposure is defined as contact with environmental tobacco smoke^23^.

### Statistical analysis

To analyze the deaths and DALYs attributable to tobacco, we employed the standard GBD comparative risk assessment framework. We first estimated the tobacco-attributable burden using population attributable fractions (PAFs), calculated as P(RR-1)/[P(RR-1)+1], where P represents the prevalence of tobacco exposure from the GBD 2021 database and RR is the relative risk of lung cancer mortality/morbidity associated with tobacco use derived from systematic reviews and meta-analyses. The theoretical minimum risk exposure level was set as no tobacco use. We then performed an age-period-cohort analysis to examine the temporal trends across birth cohorts, time periods, and age groups. This widely used epidemiological method helps decompose long-term trends into these three time-related components while accounting for their potential interactions. The final tobacco-attributable deaths and DALYs were calculated by multiplying the PAFs by the total number of lung cancer deaths and DALYs, respectively. The uncertainty intervals (UIs) were calculated using a simulation-based approach that incorporates multiple sources of uncertainty, including sampling error, model estimation, and model specification. Following GBD methodology, we generated 1000 draws from the posterior distribution of each estimate, with the 2.5 and 97.5 percentiles of these draws defining the 95% UI. This approach provides a more comprehensive assessment of uncertainty compared to conventional confidence intervals by considering various sources of variability in the estimation process.

The age-period-cohort model was used to study the trends in lung cancer burden across different age groups, periods, and birth cohorts. This model helps identify age-related biological and social factors affecting disease burden trends and can explain the trends in disease burden over time^25-27^. The age-period-cohort model is based on the log-linear Poisson model and can estimate the cumulative effects produced by changes in age, period, and birth cohort^28,29^. In the typical age-period-cohort model, age and period intervals are equal, and according to the data provided by GBD, we grouped ages (50–54, 55–59, …, 90–94, ≥95 years) and periods (1990–1994, 1995–1999, …, 2010–2014, 2015–2021) in consecutive 5-year intervals. Due to the limited data on tobacco-attributable lung cancer burden in people <50 years and between 2019–2021, we analyzed the population aged ≥50 years, and combined the years 2015–2021. Additionally, the birth year samples were grouped into consecutive 5-year intervals, covering 1898–1902 (median: 1900) to 1963–1967 (median: 1965). Net drift was used to represent the overall temporal trend in lung cancer burden, calculated after accounting for age, period, and cohort effects^30^.

To investigate the temporal trends in tobacco-related lung cancer burden between 1990 and 2021, we employed a joinpoint regression model. Two key standardized measures were analyzed: the age-standardized mortality rate (ASMR) and age-standardized disability-adjusted life year rate (ASDR). The annual average percentage change (AAPC) in these rates is a summary measure of the trend over a fixed interval, calculated as a weighted average of the annual percentage change (APC). The AAPC value and its corresponding 95% confidence interval (CI) reflect the overall trends in ASMR and ASDR. The specific method for establishing the joinpoint regression model has been reported in other literature^31^. All statistical analyses were performed using R software (version 4.3.1).

## RESULTS

### Trends in tobacco-related lung cancer burden in the Western Pacific Region from 1990 to 2021

**Figure 1:**
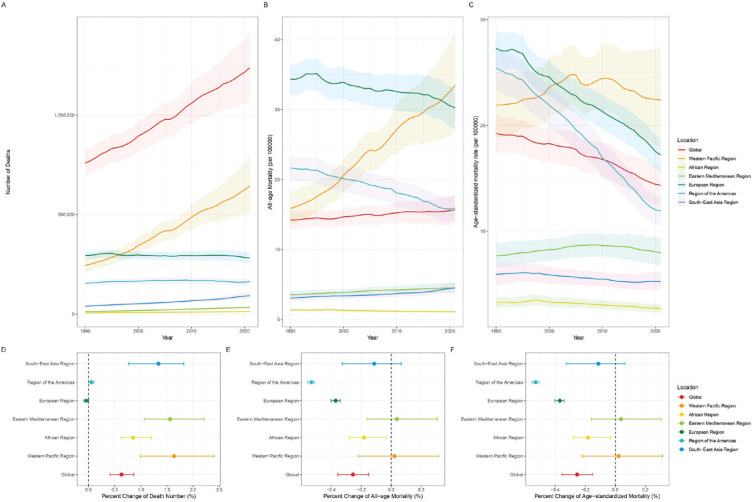
Temporal trends of the number of deaths, all-age mortality, and age-standardized mortality from tobacco-related lung cancer for both sexes combined among the six WHO regions from 1990 to 2021: A) Deaths; B) All-age mortality; C) Age-standardized mortality (solid lines and shaded areas indicate the number or rate of deaths and 95% UI); D) Percent change in number of deaths; E) Percent change of all-age mortality; F) Percent change of age-standardized mortality

From 1990 to 2021, the global number of lung cancer deaths attributable to tobacco continued to rise. In 2021, the total number of lung cancer deaths attributable to tobacco worldwide reached 1238.7 (95% UI: 1075.7–1423.1) thousand people, an increase of 63.25% (95% UI: 40.87–86.67) compared to 1990. Among the 6 regions of WHO, the Western Pacific Region ranked first in tobacco-related lung cancer deaths in 2021, with a total of 644.5 (95% UI: 517.9–793.8) thousand deaths, far exceeding the second-ranked European Region [total deaths 282.4 (95% UI: 254.0–308.4) thousand]. The tobacco-related lung cancer deaths in the Western Pacific Region accounted for more than half of all lung cancer deaths attributable to tobacco worldwide; the lowest number of deaths was in the African Region [total deaths 13.0 (95% UI: 10.9–15.2) thousand]. The number of lung cancer deaths attributable to tobacco in the Western Pacific Region in 2021 increased by 163.42% (95% UI: 99.29–239.94) compared to 1990, which is 2.6 times the global average growth rate (Table 1, Figure 1).

**Table 1:** Global trends in tobacco-related lung cancer death burden from 1990–2021

	*Sex*	*Deaths*		*All-age mortality*		*Age-standardized mortality*		
		*Number in 2021^a^* *n*	*Percent change* *1990–2021* *%*	*Rate in 2021 per* *100000*	*Percent change* *1990–2021* *%*	*Rate in 2021 per* *100000*	*Percent change* *1990–2021* *%*	*Net drift of mortality^b^* *% per year*
**Global**								
**Tobacco**	Both	1238652.2 (1075691.7–1423118.1)	63.25 (40.87–86.67)	15.7 (13.6–18.0)	10.33 (-4.79–26.17)	14.3 (12.5–16.5)	-25.46 (-35.53– -14.96)	-1.26 (-1.35 – -1.18)
	Female	249762.5 (196052.7–306054.2)	91.39 (70.25–113.53)	6.4 (5–7.8)	28.88 (14.65–43.8)	5.4 (4.2–6.6)	-13.17 (-22.59 – -3.24)	-0.97 (-1.05 – -0.88)
	Male	988889.7 (852907–1142312.5)	57.4 (31.88–84.15)	25 (21.5–28.9)	6.77 (-10.54–24.91)	25 (21.6–28.9)	-29.03 (-40.25– -17.35)	-1.41 (-1.5 – -1.31)
**Smoking**	Both	1195795.6 (1054669.9–1359222.6)	62.05 (39.75–85.51)	15.2 (13.4–17.2)	9.53 (-5.54–25.38)	13.8 (12.2–15.7)	-26.03 (-36.03 – -15.53)	-1.17 (-1.23 – -1.11)
	Female	220125.1 (187188.7–255058.7)	88.03 (68.7–108.75)	5.6 (4.8–6.5)	26.62 (13.61–40.58)	4.7 (4.0–5.5)	-14.93 (-23.68 – -5.7)	-0.96 (-1.04 – -0.87)
	Male	975670.6 (843775.9–1123054.5)	57.15 (31.66–83.99)	24.6 (21.3–28.4)	6.6 (-10.69–24.8)	24.7 (21.4–28.4)	-29.16 (-40.4 – -17.53)	-1.31 (-1.38 – -1.25)
**Secondhand smoke**	Both	97910.8 (11955.2–184912.9)	69.93 (45.22–96.94)	1.2 (0.2–2.3)	14.85 (-1.85–33.11)	1.1 (0.1–2.1)	-21.5 (-32.76 – -9.09)	-1.11 (-1.19 – -1.03)
	Female	41062.7 (5535.9–78059.2)	96.9 (60.23–139.01)	1 (0.1–2.0)	32.6 (7.9–60.95)	0.9 (0.1–1.7)	-9.17 (-25.89–10.19)	-0.83 (-0.9 – -0.75)
	Male	56848.1 (6655–109070.9)	54.63 (25.12–89.57)	1.4 (0.2–2.8)	4.89 (-15.13–28.59)	1.4 (0.2–2.8)	-28.65 (-42.44 – -13.1)	-1.34 (-1.43 – -1.25)
**Western Pacific Region**								
**Tobacco**	Both	644501.3 (517902.3–793760.5)	163.42 (99.29–239.94)	33.5 (26.9–41.2)	110.91 (59.57–172.18)	22.4 (18–27.5)	2.17 (-22.07–31.39)	-0.16 (-0.31 – -0.02)
	Female	106765.2 (72418–145384.3)	169.16 (107.1–246.57)	11.3 (7.7–15.4)	114.48 (65.02–176.16)	6.9 (4.7–9.4)	0.61 (-22.5–28.67)	-0.67 (-0.83 – -0.5)
	Male	537736.2 (418495.8–677706.7)	162.31 (87.86–249.91)	54.9 (42.7–69.2)	110.99 (51.11–181.46)	40.8 (31.8–51.0)	1.05 (-26.81–33.03)	-0.08 (-0.26–0.09)
**Smoking**	Both	616085.2 (496811.5–762787.7)	163.48 (96.27–242.18)	32 (25.8–39.6)	110.96 (57.15–173.98)	21.4 (17.3–26.4)	2.04 (-23.04–32.28)	-0.06 (-0.19–0.06)
	Female	84727.8 (65777.7–108708.4)	173.34 (105.71–257.09)	9 (7–11.5)	117.81 (63.92–184.54)	5.5 (4.2–7.0)	0.15 (-23.66–30.28)	-0.66 (-0.89 – -0.43)
	Male	531357.4 (413941.5–668636.7)	161.98 (87.59–249.82)	54.3 (42.3–68.3)	110.73 (50.9–181.38)	40.2 (31.5–50.3)	0.87 (-27–32.93)	-0.01 (-0.15–0.13)
**Secondhand smoke**	Both	63796.9 (7953.7–119778)	160.12 (104.9–229.09)	3.3 (0.4–6.2)	108.27 (64.05–163.49)	2.2 (0.3–4.2)	1.99 (-19.56–28.19)	-0.31 (-0.41 – -0.21)
	Female	29580.1 (4039.8–56719.8)	146.18 (84.5–223.51)	3.1 (0.4–6.0)	96.17 (47.02–157.78)	2 (0.3–3.7)	-2.86 (-26.96–28.02)	-0.72 (-0.82 – -0.62)
	Male	34216.8 (3770.1–68027.5)	173.51 (83.4–283.27)	3.5 (0.4–6.9)	120 (47.52–208.29)	2.6 (0.3–5.2)	6.04 (-27.51–47.37)	0.08 (-0.06–0.22)
**South-East Asia Region**								
**Tobacco**	Both	92980.2 (78554.9–108628.3)	133.69 (76.9–182.01)	4.5 (3.8–5.3)	47.58 (11.71–78.09)	5.3 (4.5–6.1)	-11.32 (-32.69–6.62)	-0.68 (-0.77 – -0.58)
	Female	10919.7 (7319.7–15525)	147.27 (94.37–218.45)	1.1 (0.7–1.5)	54.03 (21.08–98.37)	1.2 (0.8–1.7)	-11.57 (-30.85–13.74)	-0.77 (-0.94 – -0.59)
	Male	82060.4 (70222.3–94767.1)	131.99 (72.05–182.11)	7.8 (6.7–9.1)	48.44 (10.09–80.51)	9.7 (8.3–11.2)	-6.77 (-30.4–12.74)	-0.51 (-0.62 – -0.4)
**Smoking**	Both	89122.5 (76349.6–102523.9)	131.5 (74.46–179.08)	4.3 (3.7–5.0)	46.19 (10.17–76.24)	5 (4.3–5.8)	-12.22 (-33.71–5.7)	-0.67 (-0.76 – -0.59)
	Female	8342.9 (6501–10468.7)	136.15 (83.31–211.04)	0.8 (0.6–1.0)	47.11 (14.19–93.75)	0.9 (0.7–1.2)	-16.06 (-35.04–10.61)	-1.07 (-1.28 – -0.86)
	Male	80779.5 (69178–93171.2)	131.03 (71.36–180.28)	7.7 (6.6–8.9)	47.83 (9.65–79.34)	9.6 (8.2–11.0)	-7.17 (-30.74–12.28)	-0.46 (-0.55 – -0.38)
**Secondhand smoke**	Both	6925.2 (881.3–13348.9)	157.89 (97.46–211.2)	0.3 (0–0.6)	62.86 (24.7–96.52)	0.4 (0–0.7)	-1.54 (-24.45–18.96)	-0.38 (-0.59 – -0.17)
	Female	3018.2 (406–5956.7)	169.03 (105.82–243.17)	0.3 (0–0.6)	67.59 (28.21–113.77)	0.3 (0–0.6)	-1.4 (-23.82–24.98)	-0.41 (-0.71 – -0.11)
	Male	3907 (486.6–7472.4)	149.89 (80.2–217.28)	0.4 (0–0.7)	59.89 (15.31–103.02)	0.5 (0.1–0.9)	0.11 (-27.84–27.46)	-0.26 (-0.6–0.07)
**Region of the Americas**								
**Tobacco**	Both	163665.2 (144867.3–181593.5)	5.53 (0.13–10.34)	15.9 (14.1–17.7)	-26.4 (-30.17 – -23.05)	11.9 (10.6–13.2)	-53.08 (-55.47 – -50.96)	-2.9 (-2.99 – -2.81)
	Female	64445.9 (55838.7–73078)	35.67 (26.31–43.76)	12.3 (10.7–14.0)	-5.64 (-12.15 – -0.02)	8.6 (7.5–9.7)	-39.38 (-43.44 – -35.78)	-2.14 (-2.27 – -2)
	Male	99219.3 (88100.3–108830.8)	-7.77 (-12.47 – -3.48)	19.7 (17.5–21.6)	-35.5 (-38.78 – -32.5)	16 (14.2–17.6)	-59.68 (-61.72 – -57.73)	-3.41 (-3.52 – -3.31)
**Smoking**	Both	160605.7 (143994.9–176295.6)	5.3 (0.05–10.22)	15.6 (14–17.2)	-26.56 (-30.23 – -23.13)	11.7 (10.5–12.8)	-53.19 (-55.49 – -51.03)	-2.79 (-2.84 – -2.75)
	Female	63031.1 (55176.4–70440)	35.6 (26.22–43.78)	12 (10.5–13.5)	-5.69 (-12.22–0.0)	8.4 (7.4–9.3)	-39.43 (-43.5 – -35.82)	-1.99 (-2.06 – -1.92)
	Male	97574.6 (87958.6–105910.6)	-7.98 (-12.56 – -3.77)	19.4 (17.5–21.0)	-35.64 (-38.84 – -32.7)	15.7 (14.1–17.1)	-59.77 (-61.77 – -57.89)	-3.35 (-3.4 – -3.29)
**Secondhand smoke**	Both	7402.4 (936.8–14294.9)	-20.03 (-27.02 – -13.88)	0.7 (0.1–1.4)	-44.23 (-49.1 – -39.94)	0.5 (0.1–1.1)	-64.29 (-67.41 – -61.52)	-3.5 (-3.68 – -3.33)
	Female	2919 (375.3–5630.2)	-1.34 (-10.55–8.13)	0.6 (0.1–1.1)	-31.38 (-37.79 – -24.79)	0.4 (0.1–0.8)	-55.79 (-59.79 – -51.63)	-2.87 (-3.14 – -2.6)
	Male	4483.4 (561.3–8711.2)	-28.82 (-36.42 – -22.1)	0.9 (0.1–1.7)	-50.21 (-55.53 – -45.52)	0.7 (0.1–1.4)	-68.56 (-71.85 – -65.65)	-3.97 (-4.19 – -3.74)
**European Region**								
**Tobacco**	Both	282431.7 (254036.6–308364.2)	-4.16 (-9.5–0.47)	30.3 (27.2–33.0)	-11.88 (-16.79 – -7.62)	17.2 (15.5–18.7)	-36.96 (-40.18 – -33.96)	-1.77 (-1.88 – -1.66)
	Female	62323 (53268.2–70796.3)	69.34 (59.77–77.79)	13 (11.1–14.8)	56.56 (47.71–64.37)	6.8 (5.8–7.6)	16.64 (10.92–21.87)	0.45 (0.33–0.56)
	Male	220108.7 (199716.7–238934.9)	-14.65 (-19.68 – -9.99)	48.5 (44–52.6)	-21.97 (-26.57 – -17.71)	30.5 (27.7–33.1)	-47.63 (-50.63 – -44.89)	-2.49 (-2.61 – -2.36)
**Smoking**	Both	277107.3 (252969.6–299368.2)	-4.25 (-9.56–0.4)	29.7 (27.1–32.1)	-11.95 (-16.84 – -7.68)	16.9 (15.4–18.2)	-37 (-40.23 – -34.07)	-1.62 (-1.68 – -1.57)
	Female	59896.8 (52235.8–66486.7)	74.39 (64.46–83.88)	12.5 (10.9–13.9)	61.23 (52.04–70.0)	6.5 (5.8–7.2)	20.16 (14.04–25.92)	0.73 (0.66–0.81)
	Male	217210.4 (198942.7–234043.5)	-14.84 (-19.78 – -10.31)	47.8 (43.8–51.5)	-22.14 (-26.66 – -18)	30.1 (27.5–32.4)	-47.75 (-50.74 – -45.07)	-2.38 (-2.44 – -2.32)
**Secondhand smoke**	Both	15116.2 (1930.4–29189.8)	-20.67 (-27.5 – -13.52)	1.6 (0.2–3.1)	-27.06 (-33.33 – -20.48)	0.9 (0.1–1.8)	-47.13 (-51.74 – -42.36)	-2.17 (-2.3 – -2.05)
	Female	4145.9 (514–8055.8)	-2.06 (-12.05–6.75)	0.9 (0.1–1.7)	-9.46 (-18.69 – -1.31)	0.5 (0.1–0.9)	-31.52 (-38.56 – -25.38)	-1.31 (-1.52 – -1.1)
	Male	10970.4 (1416.4–21037.1)	-25.99 (-33.01 – -18.3)	2.4 (0.3–4.6)	-32.33 (-38.75 – -25.3)	1.5 (0.2–2.9)	-53.35 (-57.7 – -48.58)	-2.68 (-2.85 – -2.52)
**Eastern Mediterranean Region**								
**Tobacco**	Both	34114.4 (28729.6–40120.5)	155.94 (106.71–221.06)	4.5 (3.8–5.3)	27.7 (3.14–60.19)	8 (6.7–9.3)	3.78 (-15.86–30.63)	0.17 (0.06–0.29)
	Female	2555.4 (1538–3669.4)	231.72 (135.8–352.47)	0.7 (0.4–1.0)	66.21 (18.15–126.71)	1.3 (0.8–1.8)	35.26 (-3.08–85.75)	0.99 (0.61–1.36)
	Male	31559 (26886.7–37314)	151.29 (101.49–219.94)	8.1 (6.9–9.5)	24.9 (0.14–59.01)	14.2 (12.1–16.8)	4.51 (-16.23–33.08)	0.12 (-0.01–0.25)
**Smoking**	Both	32807 (28028.6–38502.3)	154.23 (105.39–220.16)	4.4 (3.7–5.1)	26.85 (2.48–59.74)	7.7 (6.5–9.0)	3.15 (-16.2–30.19)	0.19 (0.09–0.3)
	Female	1891 (1435.1–2417.3)	222.93 (127.81–348.58)	0.5 (0.4–0.7)	61.81 (14.15–124.76)	1 (0.7–1.2)	33.4 (-5.39–86.73)	0.99 (0.52–1.46)
	Male	30916 (26425.5–36322)	150.96 (101.15–219.63)	7.9 (6.8–9.3)	24.73 (-0.02–58.86)	13.9 (11.9–16.4)	4.4 (-16.3–33.03)	0.16 (0.05–0.27)
**Secondhand smoke**	Both	3059.4 (363.5–5984.2)	152.64 (102.33–219.31)	0.4 (0–0.8)	26.06 (0.95–59.32)	0.7 (0.1–1.4)	1.09 (-19.22–27.64)	0.1 (-0.21–0.42)
	Female	797.9 (93.9–1612.3)	241.59 (143.62–356.49)	0.2 (0–0.4)	71.15 (22.07–128.72)	0.4 (0–0.8)	35.18 (-2.41–81.71)	0.98 (0.39–1.58)
	Male	2261.5 (266–4434.2)	131.39 (84.05–198.76)	0.6 (0.1–1.1)	15 (-8.53–48.49)	1 (0.1–2.0)	-5.13 (-24.63–22.13)	-0.23 (-0.58–0.13)
**African Region**								
**Tobacco**	Both	13028.9 (10938.5–15159.7)	85.35 (62.57–119.93)	1.1 (0.9–1.3)	-18.3 (-28.34 – -3.06)	2.7 (2.3–3.1)	-18.05 (-27.9 – -3.12)	-1.11 (-1.32 – -0.91)
	Female	1735.8 (1272–2226)	109.69 (63.28–179.32)	0.3 (0.2–0.4)	-8.08 (-28.42–22.44)	0.7 (0.5–0.9)	-11.59 (-32.38–19.56)	-1.08 (-1.54 – -0.62)
	Male	11293.1 (9696.2–13168.8)	82.11 (59.34–117.73)	2 (1.7–2.3)	-19.27 (-29.37 – -3.48)	5 (4.3–5.8)	-15.67 (-25.77–1.03)	-1.01 (-1.25 – -0.77)
**Smoking**	Both	12534.1 (10872.9–14389.9)	84.57 (61.82–119.19)	1.1 (0.9–1.2)	-18.65 (-28.67 – -3.38)	2.6 (2.2–3.0)	-18.38 (-28.13 – -3.2)	-1.05 (-1.22 – -0.89)
	Female	1514.8 (1221.8–1834.9)	107.84 (60.26–181.7)	0.3 (0.2–0.3)	-8.89 (-29.75–23.49)	0.6 (0.5–0.8)	-12.28 (-33.58–20.63)	-1.09 (-1.53 – -0.64)
	Male	11019.2 (9588.2–12830.8)	81.77 (59.11–117.59)	1.9 (1.7–2.3)	-19.43 (-29.47 – -3.55)	4.9 (4.2–5.6)	-15.83 (-25.91–0.82)	-0.96 (-1.15 – -0.78)
**Secondhand smoke**	Both	853.6 (108.9–1627.6)	74.89 (46.82–109.46)	0.1 (0–0.1)	-22.91 (-35.28 – -7.67)	0.2 (0–0.3)	-23.09 (-35.3 – -6.77)	-1.23 (-1.76 – -0.7)
	Female	265.7 (33.9–504.3)	90.36 (47.68–149.82)	0 (0–0.1)	-16.55 (-35.26–9.51)	0.1 (0–0.2)	-20.53 (-38.86–5.56)	-1.08 (-2.17–0.02)
	Male	588 (73.4–1133)	68.69 (41.61–106.45)	0.1 (0–0.2)	-25.22 (-37.22 – -8.48)	0.3 (0–0.5)	-21.79 (-34.2 – -3.59)	-1.07 (-1.94 – -0.2)

The all-age mortality is equivalent–the crude mortality rate.

a The parentheses accompanying all Global Burden of Disease health estimates represent 95% uncertainty intervals, while the parentheses for net drift indicate 95% confidence intervals.

b The net drifts are estimates derived from the age-period-cohort model and signify the overall annual percentage change in mortality, encompassing the effects from calendar time and successive birth cohorts.

Compared to 1990, the global all-age mortality rate for lung cancer attributable to tobacco showed no significant change in 2021. The AMSR was 14.3 per 100000 population (95% UI: 12.5–16.5), with a percentage change of -25.46% (95% UI: -35.53 – -14.96%). However, in the Western Pacific Region, the all-age mortality for lung cancer attributable to tobacco in 2021 was 33.5 (95% UI: 26.9–41.2) cases per 100000 population, an increase of 110.91% (95% UI: 59.57–172.18) compared to 1990. However, there was no significant change in ASMR (Table 1, Figure 1). A similar phenomenon was observed in the DALYs, all-age DALYs, and ASDR attributable to tobacco-related lung cancer (Supplementary file: Table S1 and Figure S1). Similar phenomena were observed in subgroups classified by risk factors (smoking and secondhand smoke) and gender, with more pronounced effects in the smoking and female subgroups (Table 1, and Supplementary file Table S1).

From 1990 to 2021, the country with the highest burden of tobacco-related lung cancer in the Western Pacific region shifted from Brunei Darussalam [with ASMR and ASDR of 28 (21.5–35.7) cases and 634 (483.9–815.9) cases per 100000 population, respectively] to China [with ASMR and ASDR of 25.8 (20.2–32.6) cases and 575.2 (444.3–735.5) cases per 100000 population, respectively]. Fiji has consistently been the country with the lowest impact of tobacco-related lung cancer (Table 2).

**Table 2:** Trends in tobacco-related lung cancer burden in the Western Pacific Region from 1990–2021

*Location*	*ASMR per 10 ^5^* *n (95% UI)*		*ASDR per 10 ^5^* *n (95% UI)*	
	*1990*	*2021*	*1990*	*2021*
Australia	21.6 (19.6–23.6)	10.2 (8.7–11.9)	542.1 (492.6–588.0)	240.2 (207.7–278.2)
Brunei Darussalam	28 (21.5–35.7)	14.1 (10.8–17.5)	634 (483.9–815.9)	311.8 (240.1–391.3)
Cambodia	18 (12.8–25.5)	17.3 (12.1–23.0)	443.3 (318.7–635.8)	405.6 (286.2–534.4)
China	23.8 (19.9–28.1)	25.8 (20.2–32.6)	582.3 (476.8–696)	575.2 (444.3–735.5)
Cook Islands	21.5 (16.6–27.3)	15.2 (11.3–19.7)	499.5 (382–637.1)	353.6 (264.7–456.7)
Fiji	6 (4.8–7.4)	3.9 (2.9–5.2)	148.1 (117.5–186.8)	95 (68.3–129.6)
Japan	18.2 (16.8–19.6)	12.6 (11–14.0)	404.7 (375–433.7)	259 (231.7–285.9)
Kiribati	10.5 (8.1–12.7)	12.1 (9–16.0)	263.1 (206.1–317.6)	301 (220.8–398.8)
Lao People’s Democratic Republic	17.5 (11.1–28.8)	14.5 (10.3–19.8)	440 (276.6–727.0)	337.4 (241.3–463.5)
Malaysia	11.1 (9.2–14)	10.2 (8.3–12.2)	267 (222.2–337.2)	238 (194–282.0)
Marshall Islands	12.9 (8.1–20)	14.8 (8.9–22.7)	323.8 (195.6–499.9)	362.1 (215.6–559.7)
Micronesia (Federated States of)	16.6 (12–23.7)	17.5 (11.8–25.0)	425 (301.7–599.8)	446.9 (298.7–648.4)
Mongolia	19.3 (14.3–25.4)	14.4 (10.6–19.0)	493.5 (366.7–650.0)	361.4 (266.6–476.3)
Nauru	25.3 (12.4–36.2)	21.6 (10.8–28.6)	628 (306–897.6)	554.5 (271.3–753.4)
New Zealand	24.4 (21.7–26.9)	12.8 (11.1–14.8)	603.4 (540.2–661.7)	292.4 (256.7–335.5)
Niue	12.5 (9.3–16.2)	14.4 (10.2–19.4)	314 (235.4–405.5)	348.4 (243.1–473.1)
Palau	22.8 (16.7–30.3)	18.8 (14–24.8)	551.3 (403.2–734.0)	448.8 (330.9–598.0)
Papua New Guinea	7.8 (4.5–13.0)	7.8 (4.7–12.4)	193.2 (112.5–327.5)	191.5 (116.1–305.2)
Philippines	13 (11.3–14.9)	10.1 (8.1–12.5)	311.2 (270.6–360.1)	245.8 (192.9–306.7)
Republic of Korea	19.8 (16.9–23.0)	15.8 (12.9–19.2)	496 (422.7–575.6)	302.8 (252.4–364.9)
Samoa	6.4 (5.1–8.0)	5.9 (4.2–7.6)	159.9 (125.1–202.1)	147 (105.3–191.6)
Singapore	20.4 (17.8–23.2)	7 (5.8–8.3)	482.8 (422.2–546.9)	147.7 (125.3–172.9)
Solomon Islands	12.9 (8.6–18.7)	12.3 (9–16.5)	334.8 (213.7–492.9)	327.8 (234.9–446.4)
Tonga	20.7 (16.7–25.4)	19.6 (15–25.2)	473.5 (380.6–581.5)	447 (339.8–580.7)
Tuvalu	13.2 (9.1–20.1)	13.7 (10–19.0)	331.9 (227.3–507.5)	338 (245.7–470.6)
Vanuatu	9.6 (6.1–16.6)	7.7 (4.9–12.7)	230 (144.9–400.2)	182.9 (114.8–305.3)
Viet Nam	13.4 (10.3–17.0)	12.9 (9.6–16.1)	346.6 (262.1–441.4)	332.9 (243.4–422.1)

ASDR: age-standardized DALY rates. ASMR: age-standardized mortality rates.

### Trends in tobacco-related lung cancer burden across sociodemographic index quintiles in the Western Pacific Region

**Figure 2:**
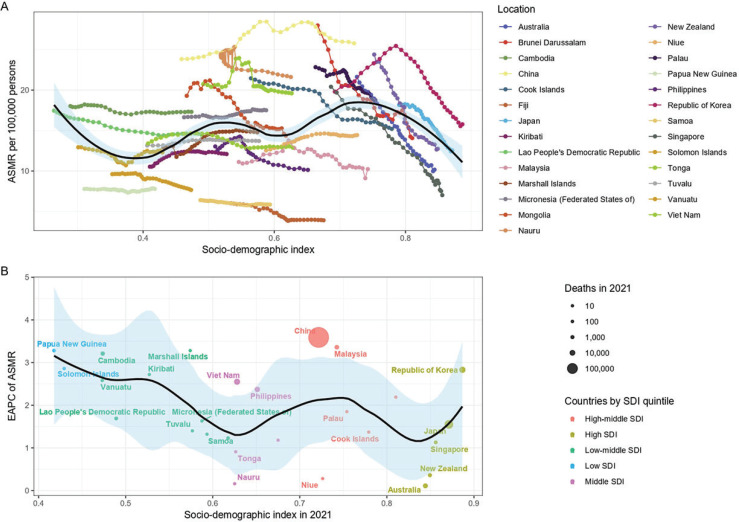
The relationship between sociodemographic index (SDI) and tobacco-related lung cancer burden in the Western Pacific Region from 1990 to 2021: A) Temporal trends of age-standardized mortality rate (ASMR) by country-specific SDI values (each line represents a country’s trajectory and the black line with shaded area indicates the expected trend with 95% CI using locally weighted scatterplot smoothing); B) The relationship between SDI values and the estimate of annual percentage change (EAPC) in ASMR

Between 1990 and 2021, in the 27 countries of the Western Pacific region, AMSR and ASDR for tobacco-related lung cancer decreased with increasing SDI values (Figure 2A, and Supplementary file Figure S2A). Overall, in the Western Pacific region, AMSR and ASDR for tobacco-related lung cancer exhibited a fluctuating decline with increasing SDI values. Similar trends were observed in both the smoking and secondhand smoke subgroups (Supplementary file Figures S3–S6).

In 2021, both AMSR and ASDR for tobacco-related lung cancer increased in the Western Pacific region (Figure 2B, and Supplementary file Figure S2B). However, with increasing SDI values, the extent of this increase diminished, as reflected by the decreasing estimate of annual percentage change (EAPC) values for AMSR and ASDR with higher SDI.

### Temporal trends in tobacco-related lung cancer burden across age groups in the Western Pacific Region

**Figure 3:**
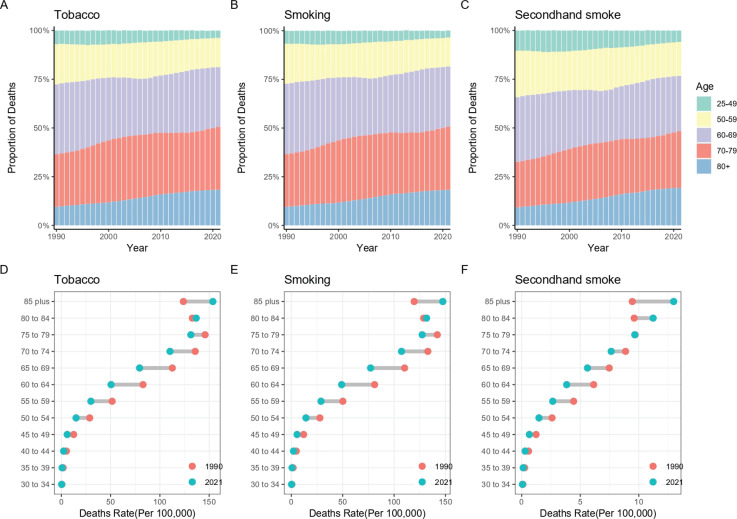
Temporal change of the mortality rate attributed to tobacco-related lung cancer across age groups in the Western Pacific Region from 1990 to 2021: The proportion of deaths by age group over time for overall tobacco exposure (A), smoking (B), and secondhand smoke exposure (C); Age-specific mortality rates (per 100000) comparing 1990 and 2021 for overall tobacco exposure (D), smoking (E), and secondhand smoke exposure (F)

From 1990 to 2021, there were significant differences in the burden of tobacco-related lung cancer across different age groups in the Western Pacific region. From 1990 to 2021, tobacco-related lung cancer deaths and DALYs primarily affected individuals aged 60–79 and 50–69 years, respectively. Additionally, the proportion of cases in older age groups (≥80 years) increased annually, while the proportion in younger age groups (<50 years) decreased annually (Figures 3A–3C, and Supplementary file Figures S7A– S7C). Compared to 1990, the changes in the burden of tobacco-related lung cancer in the Western Pacific region in 2021 were also age-related. In 2021, lung cancer mortality and DALYs due to cigarettes were lower in populations <80 years compared to 1990, but higher in populations >80 years (Figures 3D–3F, and Supplementary file Figures S7D–S7F).

### Local drift, age, period, and cohort effects on tobacco-related lung cancer burden in the Western Pacific Region

**Figure 4:**
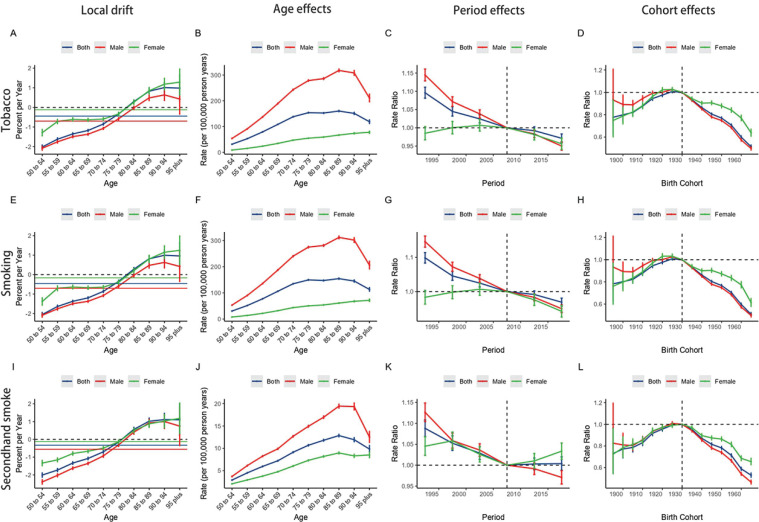
The local drifts, age effects, period effects, and cohort effects of tobacco-related lung cancer related mortality, in the Western Pacific Region from 1990 to 2021: Overall tobacco exposure showing local drift (A), age effects (B), period effects (C), and cohort effects (D); Smoking showing local drift (E), age effects (F), period effects (G), and cohort effects (H); Secondhand smoke exposure showing local drift (I), age effects (J), period effects (K), and cohort effects (L). All analyses are stratified by sex

The age-period-cohort model assessed the trends in mortality and DALYs of tobacco-attributable lung cancer from four aspects: local drift, age effect, period effect, and cohort effect. Overall, the net drift of lung cancer mortality [-1.06% (95% UI: -0.31 – -0.02) per year] and DALYs [-0.17% (95% UI: -0.32 – -0.01) per year] in the Western Pacific Region were lower than the global mortality rate [-1.26% (95% UI: -1.35 – -1.18) per year] and DALYs [-1.26% (95% UI: -1.35 – -1.17) per year] (Table 1, and Supplementary file Table S1). The local drift values for lung cancer mortality and DALYs continued to decline in individuals <80 years, while showing a persistent increase in those >85 years. Similar trends were observed in subgroups stratified by tobacco use and gender (Figures 4A, 4E and 4I, and Supplementary file Figures S8A, S8E and S8I).

The longitudinal age distribution showed that lung cancer-related mortality and DALYs initially increased and then decreased with age. A similar phenomenon was also observed in the cohort effect (Figures 4B and 4D, and Supplementary file Figures S8B and S8D).

Regarding the period effect, we observed a continuous decline in lung cancer mortality and DALYs due to tobacco, with a more pronounced decrease in males (Figure 4C, and Supplementary file Figure S8C).

### Long-term trends in the tobacco-related lung cancer burden in the Western Pacific Region from 1990 to 2021

To understand the detailed changes in lung cancer burden, the period from 1990 to 2021 was divided into six consecutive stages. From 1990 to 2021, the ASMR and ASDR for tobacco-related lung cancer globally have shown a continuous decline (Supplementary file Figure S9). However, in the Western Pacific Region, the AMSR for lung cancer exhibited an eventual increase (AAPC=0.08), while the ASDR showed a final decrease (AAPC= -0.18) (Supplementary file Figure S9). Although the trends in the ASMR and ASDR for tobacco-related lung cancer in the Western Pacific Region are divergent, both ASMR and ASDR exhibit similar patterns of change. Between 1998 and 2004, ASMR and ASDR increased at the fastest rates (APC=1.53% and 1.08%, respectively), while between 2004 and 2007, ASMR and ASDR decreased at the fastest rates (APC= -1.49% and -1.48%, respectively). Conversely, from 1990 to 1998, ASMR and ASDR increased at the slowest rates (APC=0.5% and 0.16%, respectively), and from 2018 to 2021, ASMR and ASDR decreased at the slowest rates (APC= -0.29% and -0.4%, respectively). The trend observed in the burden of lung cancer related to tobacco subgroups (smoking and secondhand smoke) shows similar patterns (Supplementary file Figures S10–S14).

## DISCUSSION

Our analyses revealed several key findings regarding tobacco-related lung cancer burden in the Western Pacific Region from 1990 to 2021. First, while global tobacco-related lung cancer deaths increased by 63.25%, the Western Pacific Region saw a more dramatic rise of 163.42%, accounting for over half of global tobacco-related lung cancer deaths by 2021. Second, although age-standardized mortality rates remained relatively stable globally, the Western Pacific Region showed an eventual increase (AAPC=0.08). Third, the burden varied by SDI level, with higher SDI countries generally showing lower rates and smaller increases over time. Fourth, age-period-cohort analysis revealed increasing burden among the elderly (>84 years), while showing declining period and cohort effects. These findings highlight the disproportionate and growing impact of tobacco-related lung cancer in the Western Pacific Region.

Lung cancer is one of the most common cancers. Smoking promotes the development and progression of lung cancer and is the most important risk factor for the disease. Smoking is highly prevalent worldwide, with the global age-standardized smoking rate for men (32.7%) being significantly higher than that for women (6.62%). Despite the implementation of global smoking bans and related policies, differences in lifestyle, cultural practices, and economic and political factors across various countries and regions have resulted in varying degrees of enforcement of these bans worldwide^32,33^. The WHO monitors the implementation of the Framework Convention on Tobacco Control through the MPOWER framework. Over the past decade, only Turkey and Brazil have fully implemented the policies outlined in MPOWER at the highest level. Since 1990, global smoking rates have declined significantly; however, the rapid growth of the population has led to an increase in the total number of smokers^15^. Two-thirds of the global smoking population is concentrated in the Western Pacific region, where some areas have a large number of smokers^15^. This will undoubtedly result in a significant burden of tobacco-related diseases in the Western Pacific region.

As shown in our study, the total number of global lung cancer deaths attributable to tobacco reached 1238.7 thousand (95% UI: 1075.7–1423.1) in 2021, representing a 63.25% (95% UI: 40.87–86.67) increase compared to 1990. While there was no significant change in the all-age mortality rate for lung cancer caused by tobacco globally, the ASMR [14.3 (95% UI: 12.5–16.5) per 100000 individuals] for tobacco-induced lung cancer decreased by 25.46% compared to 1990. We observed similar changes in global DALYs attributable to tobacco-induced lung cancer. This phenomenon can be explained by global population growth, population aging, and the implementation of smoking bans. For example, legislation and policies that prohibit smoking in public places such as restaurants, hospitals, hotels, and public transportation have mitigated the tobacco epidemic to some extent^34,35^. However, the dramatic increase in the global population and the increase in the aged population, have led to an increased overall burden of lung cancer. Therefore, the total burden of smoking-related lung cancer continues to rise globally, but the global tobacco-related lung cancer ASMR and ASDR have decreased after considering age factors. This phenomenon can reflect the results of global tobacco control efforts. However, it is worth noting that in our gender subgroup analysis, although the burden of tobacco-related lung cancer is generally higher in males than in females, the increase in lung cancer burden in females is faster, which may be related to the increase in the smoking rate among females.

Among the 6 major regions of the world defined by WHO, the Western Pacific Region has the largest population (1/4 of the global) and the highest smoking rate (about 2/3 of the world’s smokers), so the burden of tobacco-related lung cancer also far exceeds other regions. We observed that from 1990 to 2021, the increase in the burden of lung cancer attributable to tobacco in the Western Pacific Region was the most significant, with the total number of deaths and DALYs accounting for more than half of the global total, and the growth rate of deaths was 2.6 times the global level. In addition, compared with 1990, the country with the heaviest burden of tobacco-related lung cancer in the Western Pacific Region in 2021 changed from Brunei Darussalam to China. From 1990 to 2021, the overall trend in the burden of tobacco-related lung cancer shows a significant global decrease in both ASMR and ASDR. However, in the Western Pacific region, the ASMR for lung cancer has actually increased (AAPC= 0.08). It can be seen that the burden of tobacco-related lung cancer in the Western Pacific Region is not optimistic, which to some extent reflects the high prevalence of tobacco in the Western Pacific Region, the lack of implementation of smoking bans, and the development differences between countries in the Western Pacific Region. As previous studies have shown, since 1990, the global tobacco epidemic has been controlled to some extent, but the smoking rate in some countries in the Western Pacific Region has not significantly decreased^36^. This indicates the necessity of formulating more effective policies to manage the burden of tobacco-induced lung cancer in the Western Pacific Region.

The SDI is a key factor influencing the burden of disease, as it reflects disparities in the population’s baseline health status and social resources (economic, healthcare, etc.). Countries with a high SDI have better public healthcare resources and more effective methods for the diagnosis and treatment of diseases. This phenomenon is also observed in our study. In the 27 countries of the Western Pacific, the burden of tobacco-related lung cancer decreases with increasing SDI, as reflected by reductions in ASMR and ASDR. In 2021, nearly all countries in the Western Pacific experienced an increase in ASMR and ASDR of lung cancer attributable to tobacco. However, with increasing SDI, the magnitude of the increase in ASMR and ASDR has diminished. This is reflected in the decreasing EAPC values of ASMR and ASDR with higher SDI values.

Tobacco-related lung cancer ASMR and ASDR predominantly affect different age groups, which can be explained by the different types of indicators used for observation. From 1990 to 2021, the proportion of tobacco-related lung cancer ASMR and ASDR has gradually increased among the elderly population (>80 years). Similarly, in 2021, the ASMR and ASDR for tobacco-related lung cancer in individuals <80 years were lower than in 1990, while in those >80 years, they were higher than in 1990. In the age-time-cohort model, we also observed an increase in the burden of tobacco-related lung cancer among the elderly population (>84 years). Additionally, the age effect analysis shows that tobacco-related lung cancer mortality increases with age. These phenomena are consistent with the aging characteristics of the population in the Western Pacific region^20^.

In the period and cohort effects, a downward trend in the ASMR and ASDR for tobacco-related lung cancer has been observed. This trend may be attributed to improvements in living standards and healthcare conditions resulting from social development.

### Limitations

Our study has several important limitations. First, as a secondary analysis based on the GBD 2021 database, our research involves the inherent limitations of GBD methodology, including potential uncertainties in data collection, modeling assumptions, and estimation procedures. Second, our analysis does not differentiate between types of active smoking (e.g. cigarettes, cigars, pipes) or intensity of smoking, which could provide more detailed insights into risk patterns. Third, while we included secondhand smoke exposure in our analysis, the full impact of passive smoking may be underestimated due to challenges in exposure assessment and attribution. Fourth, the assumptions underlying our regression analyses and EAPC estimations, such as linearity and time trends, may not fully capture complex temporal patterns. Finally, our analysis is based on ecological and crosssectional data, which limits our ability to establish direct causal relationships. The GBD database may not fully capture all relevant socioeconomic, cultural, and healthcare system factors that could influence tobacco use patterns and lung cancer outcomes across different populations.

## CONCLUSIONS

This study uses the latest GBD 2021 dataset to reveal changes in the burden of tobacco-related lung cancer in the Western Pacific region from 1990 to 2021. The results indicate that the burden of lung cancer attributable to tobacco remains high in the Western Pacific region compared to the global level.

## Supplementary Material



## Data Availability

The data supporting this research can be found in the Supplementary file. Further inquiries can be directed to the corresponding author.
