# Supplementary material for: Tobacco-related lung cancer burden in the Western Pacific Region from 1990 to 2021: An age-period-cohort analysis from the Global Burden of Disease Study
Source: Tob Induc Dis. 2025 Mar 13;23:10.18332/tid/201970. doi: 10.18332/tid/201970 (PMC11905381; doi:10.18332/tid/201970)
Supplement: Supplementary file 1 [file TID-23-30-s1.pdf]

Supplemental

- Supplementary Table S1 The trends in the tobacco-related lung cancer DALYs burden in the Global from 1990 to 2021
- Supplementary Figure S1 Temporal trends of the numbers of DALYs, all-age DALYs, and ASDR for tobacco-related lung cancer for both sexes combined among the six WHO regions from 1990 to 2021.
- Supplementary Figure S2 The relationship between SDI levels and the age-standardized DALYs for tobacco-related lung cancer in the Western Pacific Region from 1990 to 2021
- Supplementary Figure S3 The relationship between SDI levels and the age-standardized death rate for smoking-related lung cancer in the Western Pacific Region from 1990 to 2021
- Supplementary Figure S4 The relationship between SDI levels and the age-standardized death rate for secondhand smoke-related lung cancer in the Western Pacific Region from 1990 to 2021
- Supplementary Figure S5 The relationship between SDI levels and the age-standardized DALYs for smoking-related lung cancer in the Western Pacific Region from 1990 to 2021
- Supplementary Figure S6 The relationship between SDI levels and the age-standardized DALYs for secondhand smoke-related lung cancer in the Western Pacific Region from 1990 to 2021
- Supplementary Figure S7 The temporal change of the DALYs rate attributed to tobacco-related lung cancer across age groups in the Western Pacific Region from 1990 to 2021
- Supplementary Figure S8 The local drifts, age effects, period effects, and cohort effects of tobacco-related lung cancer related DALYs rate in the Western Pacific Region from 1990 to 2021.
- Supplementary Figure S9 The joinpoint regression analysis of the tobacco-related lung cancer burden in the Western Pacific Region from 1990 to 2021.
- Supplementary Figure S10 The joinpoint regression analysis of the tobacco-related lung cancer burden in Global from 1990 to 2021.
- Supplementary Figure S11 The joinpoint regression analysis of the smoking-related lung cancer burden in the Western Pacific Region from 1990 to 2021.
- Supplementary Figure S12 The joinpoint regression analysis of the secondhand smoke-related lung cancer burden in the Western Pacific Region from 1990 to 2021.
- Supplementary Figure S13 The joinpoint regression analysis of the smoking-related lung cancer burden in Global from 1990 to 2021.
- Supplementary Figure S14 The joinpoint regression analysis of the secondhand smoke-related lung cancer burden in Global from 1990 to 2021.

Supplementary Table S1 The trends in the tobacco-related lung cancer DALYs burden in the Global from 1990 to 2021

	Sex	DALYs		All-age DALYs		ASDR		
		Number in 2021[a], n	Change of numbers 1990-2021, %	Rate in 2021, per 100000	Percent change 1990-2021, %	Rate in 2021, per 100000	Percent change 1990-2021, %	Net drift of DALYs[b], % per year
	Global							
Tobacco	Both	28768021.6 (24948938 to 33061214.1)	44.21 (24.6 to 65.81)	364.6 (316.2 to 419)	-2.53 (-15.79 to 12.07)	327.8 (284.2 to 376.8)	-32.45 (-41.6 to -22.45)	-1.26 (-1.35 to -1.17)
Tobacco	Female	5516414.7 (4306882 to 6740465.2)	69.37 (51.67 to 89.02)	140.3 (109.5 to 171.4)	14.06 (2.14 to 27.29)	119.3 (93 to 145.9)	-21.1 (-29.3 to -11.83)	-0.96 (-1.02 to -0.89)
Tobacco	Male	23251607 (20027487.3 to 26946174.4)	39.3 (15.98 to 64.11)	587.3 (505.8 to 680.6)	-5.51 (-21.33 to 11.32)	561.6 (484 to 650.2)	-35.05 (-45.77 to -23.75)	-1.4 (-1.51 to -1.3)
Smoking	Both	27713689.2 (24404593.2 to 31596581.2)	43.17 (23.41 to 64.74)	351.2 (309.3 to 400.4)	-3.23 (-16.59 to 11.35)	315.7 (278.2 to 359.9)	-33 (-42.18 to -22.98)	-1.17 (-1.25 to -1.08)
Smoking	Female	4780176.6 (4165305.6 to 5493258.4)	66.35 (50.21 to 83.37)	121.6 (105.9 to 139.7)	12.03 (1.16 to 23.49)	103.1 (89.9 to 118.4)	-22.86 (-30.19 to -15.1)	-0.95 (-1.02 to -0.88)

	Male	22933512.6 (19793991.4 to 26526587.7)	39.13 (15.84 to 63.97)	579.2 (499.9 to 670)	-5.62 (-21.43 to 11.22)	553.8 (479.3 to 639.6)	-35.16 (-45.86 to -23.86)	-1.31 (-1.41 to -1.22)
Secondhand smoke	Both	2355866 (290210.9 to 4442996.3)	47.35 (26.29 to 71.3)	29.9 (3.7 to 56.3)	-0.41 (-14.65 to 15.78)	26.9 (3.3 to 50.8)	-29.89 (-39.75 to -18.58)	-1.1 (-1.21 to -1)
	Female	996309.4 (135988.2 to 1907079)	72.39 (40.55 to 109.97)	25.3 (3.5 to 48.5)	16.09 (-5.35 to 41.4)	21.8 (3 to 41.8)	-17.88 (-32.85 to -0.17)	-0.82 (-0.9 to -0.74)
	Male	1359556.6 (158640 to 2596453.4)	33.17 (7.72 to 62.1)	34.3 (4 to 65.6)	-9.67 (-26.93 to 9.96)	32.9 (3.8 to 62.8)	-36.63 (-48.59 to -22.9)	-1.34 (-1.47 to -1.21)
Western Pacific Region								
Tobacco	Both	14615025.5 (11614074.6 to 18203951.7)	125.82 (68.73 to 194.98)	759.1 (603.2 to 945.5)	80.8 (35.1 to 136.18)	503.9 (400.8 to 627.4)	-5.8 (-29.35 to 22.66)	-0.17 (-0.32 to -0.01)
	Female	2255437.7 (1496664.7 to 3154639.2)	129.29 (74.3 to 200.97)	238.4 (158.2 to 333.4)	82.71 (38.89 to 139.83)	149 (97.5 to 209.1)	-6.82 (-29.17 to 21.17)	-0.68 (-0.81 to -0.55)

	Male	12359587.8 (9591289.3 to 15733255.4)	125.19 (59.27 to 206.62)	1262.4 (979.7 to 1607)	81.14 (28.11 to 146.63)	893.5 (694.6 to 1133.3)	-5.99 (-32.91 to 26.71)	-0.09 (-0.28 to 0.1)
	Both	13927994.7 (11085580.5 to 17403470)	126.34 (66.41 to 198.33)	723.4 (575.8 to 903.9)	81.23 (33.24 to 138.86)	479.4 (381.6 to 597.9)	-5.9 (-30.57 to 23.61)	-0.06 (-0.23 to 0.1)
Smoking	Female	1714642.8 (1322771.1 to 2215012.2)	135.4 (77.21 to 211.31)	181.2 (139.8 to 234.1)	87.57 (41.21 to 148.07)	111.7 (85.9 to 144)	-6.94 (-29.67 to 22.46)	-0.67 (-0.86 to -0.48)
	Male	12213351.9 (9468100.2 to 15509922.7)	125.13 (59.1 to 206.71)	1247.5 (967.1 to 1584.2)	81.09 (27.98 to 146.71)	882.3 (687.6 to 1116.4)	-6.11 (-33.09 to 26.64)	-0.01 (-0.22 to 0.19)
	Both	1485063.6 (187691.8 to 2776391.9)	118.99 (71.75 to 179.31)	77.1 (9.7 to 144.2)	75.34 (37.52 to 123.63)	51.9 (6.6 to 97.3)	-6.07 (-26.11 to 19.4)	-0.31 (-0.44 to -0.18)
Secondhand smoke	Female	701642.3 (96388.6 to 1346530.9)	108.32 (54.07 to 178.65)	74.1 (10.2 to 142.3)	66 (22.77 to 122.04)	47.8 (6.6 to 91.9)	-10.06 (-33.01 to 19.99)	-0.72 (-0.83 to -0.61)

	South-East Asia Region	Male	783421.4 (86527.8 to 1553687.7)	129.51 (52.81 to 226.81)	80 (8.8 to 158.7)	84.61 (22.92 to 162.88)	57.3 (6.3 to 113.4)	-2.53 (-34.44 to 37.14)	0.07 (-0.12 to 0.27)
		Both	2383482 (1998245.5 to 2789798.6)	115.81 (62.98 to 160.25)	115.5 (96.8 to 135.2)	36.29 (2.92 to 64.35)	125.9 (105.9 to 147.4)	-14.48 (-35.34 to 3.2)	-0.68 (-0.8 to -0.56)
Tobacco		Female	265901.8 (171191.5 to 387043)	124.01 (75.63 to 191.12)	26.1 (16.8 to 38)	39.54 (9.4 to 81.35)	27.7 (18.1 to 40.1)	-15.71 (-34.26 to 9.2)	-0.78 (-0.9 to -0.67)
		Male	2117580.2 (1810202.9 to 2453529.6)	114.82 (58.52 to 163.37)	202.5 (173.1 to 234.6)	37.46 (1.43 to 68.52)	231.3 (198.3 to 267.4)	-10.48 (-33.46 to 9.19)	-0.51 (-0.65 to -0.38)
Smoking		Both	2273943.3 (1947412.3 to 2622069.5)	113.58 (60.8 to 158.3)	110.2 (94.4 to 127.1)	34.88 (1.55 to 63.12)	120.3 (103 to 138.7)	-15.47 (-36.32 to 1.99)	-0.67 (-0.8 to -0.55)
		Female	192838.1 (149246.2 to 243463)	110.86 (64.14 to 178.51)	18.9 (14.7 to 23.9)	31.35 (2.25 to 73.49)	20.4 (15.9 to 25.7)	-21.53 (-38.92 to 3.31)	-1.08 (-1.23 to -0.93)

	Male	2081105.2 (1785092.5 to 2411889.5)	113.83 (57.87 to 161.51)	199 (170.7 to 230.6)	36.82 (1.02 to 67.33)	227.5 (194.9 to 262.9)	-10.92 (-33.87 to 8.57)	-0.47 (-0.61 to -0.33)
Secondhand smoke	Both	187226.5 (23689 to 359492.3)	140.79 (83.3 to 191.49)	9.1 (1.1 to 17.4)	52.06 (15.76 to 84.08)	9.7 (1.2 to 18.7)	-3.17 (-26 to 17.21)	-0.38 (-0.48 to -0.28)
	Female	83627.1 (11329.7 to 166465.2)	149.3 (91.66 to 222.07)	8.2 (1.1 to 16.4)	55.29 (19.39 to 100.63)	8.4 (1.1 to 16.7)	-2.66 (-25.3 to 25.33)	-0.43 (-0.52 to -0.34)
	Male	103599.3 (12853.4 to 197715.9)	134.33 (68.81 to 196.13)	9.9 (1.2 to 18.9)	49.94 (8.02 to 89.48)	11.2 (1.4 to 21.3)	-2.24 (-29.43 to 23.82)	-0.3 (-0.45 to -0.14)
Region of the Americas								
Tobacco	Both	3662478.2 (3278872 to 4015925.6)	-5.89 (-10.39 to -1.96)	356.7 (319.3 to 391.1)	-34.37 (-37.51 to -31.63)	270 (241.7 to 296)	-57.92 (-59.92 to -56.15)	-2.89 (-2.96 to -2.82)
	Female	1424703.6 (1251525.6 to 1592405.5)	19.83 (12.89 to 26.35)	272.3 (239.2 to 304.4)	-16.66 (-21.48 to -12.13)	195.8 (172.4 to 218.5)	-46.3 (-49.36 to -43.41)	-2.13 (-2.21 to -2.05)

	Male	2237774.5 (2012598.2 to 2439612.5)	-17.21 (-21.07 to -13.52)	444.3 (399.6 to 484.4)	-42.09 (-44.8 to -39.52)	356.8 (320.5 to 389.3)	-63.28 (-65.01 to -61.64)	-3.42 (-3.49 to -3.34)
Smoking	Both	3591664.8 (3262580.3 to 3897482.1)	-6.12 (-10.44 to -2.28)	349.8 (317.7 to 379.5)	-34.53 (-37.54 to -31.85)	264.6 (240.4 to 287.1)	-58.04 (-59.95 to -56.34)	-2.79 (-2.83 to -2.74)
	Female	1392089.3 (1243343.9 to 1535672.7)	19.73 (12.85 to 26.26)	266.1 (237.6 to 293.5)	-16.73 (-21.51 to -12.18)	191.2 (171.2 to 210.6)	-46.39 (-49.45 to -43.49)	-1.98 (-2.04 to -1.93)
	Male	2199575.5 (2001630.1 to 2375374.9)	-17.41 (-21.23 to -13.78)	436.7 (397.4 to 471.6)	-42.23 (-44.91 to -39.7)	350.5 (318.8 to 378.9)	-63.39 (-65.08 to -61.79)	-3.35 (-3.4 to -3.3)
Secondhand smoke	Both	177646 (22276.1 to 343541.7)	-28.6 (-34.69 to -23.41)	17.3 (2.2 to 33.5)	-50.2 (-54.45 to -46.59)	13.3 (1.7 to 25.8)	-67.68 (-70.42 to -65.38)	-3.5 (-3.59 to -3.41)
	Female	69655.7 (8813.4 to 135471.9)	-12.85 (-20.3 to -4.77)	13.3 (1.7 to 25.9)	-39.39 (-44.57 to -33.77)	9.8 (1.2 to 19.2)	-60.39 (-63.88 to -56.71)	-2.87 (-2.94 to -2.79)

	European Region	Male	107990.3 (13462.7 to 207833.4)	-36.05 (-42.68 to -30.18)	21.4 (2.7 to 41.3)	-55.28 (-59.91 to -51.17)	17.3 (2.2 to 33.4)	-71.25 (-74.22 to -68.69)	-3.97 (-4.07 to -3.87)
Tobacco		Both	6658084.7 (6015307.6 to 7233645.3)	-14.82 (-19.18 to -10.88)	713.3 (644.4 to 774.9)	-21.68 (-25.69 to -18.06)	427.8 (386.9 to 464.1)	-41.98 (-44.87 to -39.39)	-1.77 (-1.86 to -1.68)
		Female	1433260.6 (1236882.6 to 1608676.3)	57.86 (50.04 to 65.14)	299 (258 to 335.6)	45.95 (38.71 to 52.67)	170 (147.7 to 190.9)	12.02 (6.75 to 17.11)	0.46 (0.39 to 0.54)
		Male	5224824.1 (4765860.2 to 5642466.6)	-24.37 (-28.67 to -20.56)	1150.6 (1049.5 to 1242.5)	-30.86 (-34.79 to -27.37)	737.8 (673 to 797.3)	-51.04 (-53.84 to -48.6)	-2.49 (-2.58 to -2.39)
Smoking		Both	6532814.3 (6000352.7 to 7008750.7)	-14.87 (-19.25 to -11.13)	699.9 (642.8 to 750.9)	-21.72 (-25.75 to -18.28)	419.4 (385.4 to 448.9)	-42.03 (-44.91 to -39.53)	-1.62 (-1.69 to -1.55)
		Female	1376413.8 (1228855 to 1507256)	62.86 (54.49 to 70.67)	287.2 (256.4 to 314.5)	50.56 (42.82 to 57.79)	162.9 (145.9 to 178)	15.54 (9.76 to 21.18)	0.74 (0.68 to 0.81)

		Male	5156400.5 (4748935.5 to 5532241.9)	-24.49 (-28.79 to -20.74)	1135.5 (1045.8 to 1218.3)	-30.96 (-34.89 to -27.54)	727.9 (670.4 to 781.6)	-51.12 (-53.93 to -48.69)	-2.38 (-2.45 to -2.31)
	Secondhand smoke	Both	378371.6 (48413.7 to 728993.3)	-29.31 (-35.47 to -22.44)	40.5 (5.2 to 78.1)	-35 (-40.66 to -28.68)	25.1 (3.2 to 48.4)	-50.94 (-55.31 to -46.23)	-2.17 (-2.24 to -2.1)
	Secondhand smoke	Female	102750 (12678.3 to 198818.5)	-8.54 (-18.27 to -0.16)	21.4 (2.6 to 41.5)	-15.44 (-24.44 to -7.7)	13 (1.6 to 25)	-33.51 (-40.5 to -27.4)	-1.3 (-1.39 to -1.22)
	Secondhand smoke	Male	275621.6 (35735.4 to 529539.1)	-34.82 (-41.13 to -27.89)	60.7 (7.9 to 116.6)	-40.41 (-46.18 to -34.08)	39.6 (5.1 to 76.1)	-56.69 (-60.84 to -52.21)	-2.68 (-2.75 to -2.61)
Eastern Mediterranean Region									
	Tobacco	Both	916428.5 (768035.6 to 1085175.8)	155.28 (107.46 to 221.8)	121.7 (102 to 144.1)	27.38 (3.51 to 60.56)	190.6 (160.8 to 224.7)	0.74 (-18.54 to 26.7)	0.18 (0.1 to 0.25)
	Tobacco	Female	68436.9 (40307.9 to 100257)	215.95 (124.34 to 328.99)	18.9 (11.1 to 27.7)	58.31 (12.4 to 114.95)	29.6 (17.8 to 42.7)	24.67 (-11.41 to 69.89)	1.01 (0.86 to 1.16)

	Male	847991.6 (717455 to 1006101.1)	151.39 (102.26 to 219.33)	216.9 (183.5 to 257.3)	24.94 (0.52 to 58.71)	340 (289 to 402.6)	1.29 (-18.74 to 28.74)	0.12 (0.05 to 0.2)
	Both	878217 (749257.6 to 1031465.4)	153.48 (105.35 to 219.02)	116.6 (99.5 to 137)	26.47 (2.46 to 59.17)	183.2 (156.5 to 215.3)	0.12 (-19.05 to 25.81)	0.19 (0.11 to 0.27)
Smoking	Female	48632.9 (36929.3 to 62284.6)	204.01 (114.08 to 322.92)	13.4 (10.2 to 17.2)	52.32 (7.26 to 111.91)	21.7 (16.6 to 27.7)	21.33 (-14.68 to 68.29)	0.97 (0.77 to 1.18)
	Male	829584.1 (707961 to 973375.6)	151.03 (102.02 to 219.1)	212.2 (181.1 to 249)	24.76 (0.41 to 58.59)	333 (284.1 to 391.2)	1.19 (-18.75 to 28.7)	0.16 (0.08 to 0.24)
	Both	86063.9 (10272 to 168271.9)	153.99 (105.1 to 219.7)	11.4 (1.4 to 22.3)	26.73 (2.34 to 59.51)	17.3 (2.1 to 33.8)	-1.21 (-20.37 to 24.68)	0.09 (0.01 to 0.17)
Secondhand smoke	Female	23337.3 (2792.3 to 46812.9)	232.31 (134.4 to 340.52)	6.4 (0.8 to 12.9)	66.5 (17.45 to 120.72)	9.4 (1.1 to 19)	28.33 (-8.52 to 70.87)	1.06 (0.89 to 1.23)
	Male	62726.6	133.51 (85.28 to 203.16)	16 (1.9 to 31.5)	16.06 (-7.92 to 50.67)	24.5 (2.9 to 48.1)	-7.2 (-26.23 to 19.87)	-0.21 (-0.3 to -0.11)

		(7448.7 to 123351.8)						
	African Region							
	Both	355879.2 (299592.5 to 415787.3)	82.33 (59.19 to 116.47)	30.8 (25.9 to 36)	-19.63 (-29.83 to -4.58)	65.6 (55.2 to 76.3)	-20.33 (-30.21 to -5.35)	-1.13 (-1.28 to -0.98)
Tobacco	Female	44537.5 (32396.7 to 56975)	101.24 (57.77 to 168.04)	7.6 (5.5 to 9.7)	-11.78 (-30.84 to 17.5)	16.1 (11.8 to 20.6)	-14.94 (-33.38 to 13.79)	-1.06 (-1.35 to -0.76)
	Male	311341.6 (266332.3 to 366089)	79.91 (56.37 to 115.54)	54.7 (46.8 to 64.3)	-20.25 (-30.68 to -4.45)	121.7 (104.6 to 142.2)	-17.37 (-27.86 to -1)	-1.04 (-1.2 to -0.89)
	Both	341364.8 (297114.8 to 395715.7)	81.55 (58.47 to 116.22)	29.5 (25.7 to 34.2)	-19.97 (-30.15 to -4.69)	63.1 (54.8 to 72.6)	-20.61 (-30.55 to -5.63)	-1.06 (-1.22 to -0.9)
Smoking	Female	38174.8 (30807 to 46135.7)	98.91 (54.37 to 170.08)	6.5 (5.3 to 7.9)	-12.81 (-32.33 to 18.39)	14 (11.3 to 17)	-15.59 (-34.26 to 14.98)	-1.12 (-1.45 to -0.78)
	Male	303190.1 (263635.5 to 354255.6)	79.58 (56.14 to 115.13)	53.3 (46.3 to 62.3)	-20.4 (-30.78 to -4.64)	118.7 (103.4 to 138.6)	-17.49 (-27.91 to -1.17)	-0.96 (-1.12 to -0.8)

Secondhand smoke	Both	24558.7 (3163.1 to 47109.3)	71.5 (43.58 to 106.15)	2.1 (0.3 to 4.1)	-24.4 (-36.71 to -9.13)	4.3 (0.6 to 8.3)	-25.84 (-37.63 to -11.15)	-1.21 (-1.37 to -1.06)
	Female	7535.4 (964.1 to 14256)	85.66 (42.13 to 140.66)	1.3 (0.2 to 2.4)	-18.61 (-37.7 to 5.5)	2.5 (0.3 to 4.8)	-23.4 (-40.74 to 0.19)	-1.04 (-1.26 to -0.81)
	Male	17023.4 (2142.5 to 32988)	65.9 (37.14 to 101.82)	3 (0.4 to 5.8)	-26.46 (-39.21 to -10.53)	6.4 (0.8 to 12.3)	-24.37 (-36.79 to -7.68)	-1.19 (-1.39 to -0.98)

The all-age mortality is equivalent to the crude mortality rate.

a The parentheses accompanying all Global Burden of Disease health estimates represent 95% uncertainty intervals, while the parentheses for net drift indicate 95% confidence intervals.

b The net drifts are estimates derived from the age–period–cohort model and signify the overall annual percentage change in mortality, encompassing the effects from calendar time and successive birth cohorts.

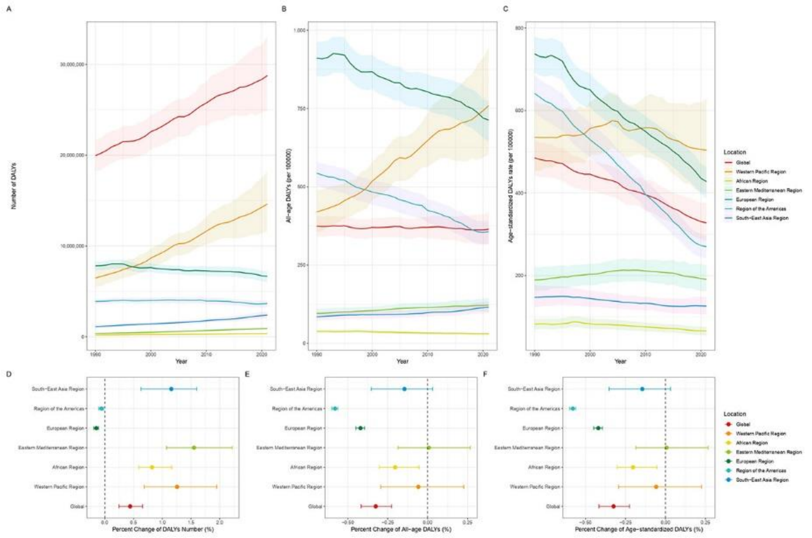

Supplementary Figure S1 Temporal trends of the numbers of DALYs, all-age DALYs, and ASDR for tobacco-related lung cancer for both sexes combined among the six WHO regions from 1990 to 2021. (A-C) Temporal trends of the numbers of DALYs, all-age DALYs rates, and ASDR for tobacco-related lung cancer from 1990 to 2021. The solid lines and shaded areas indicate the number or rate of DALYs and the corresponding 95% uncertainty intervals. (D-F) The % change of the numbers of DALYs, all-age DALYs rates, and ASDR for tobacco-related lung cancer among the six WHO regions from 1990 to 2021.

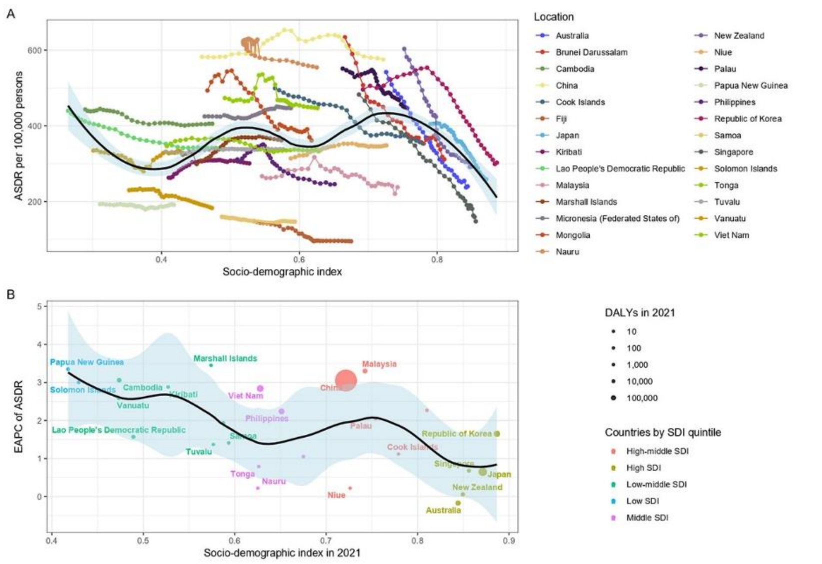

Supplementary Figure S2 The relationship between SDI levels and the age-standardized DALYs for tobacco-related lung cancer in the Western Pacific Region from 1990 to 2021

SDI: sociodemographic index.

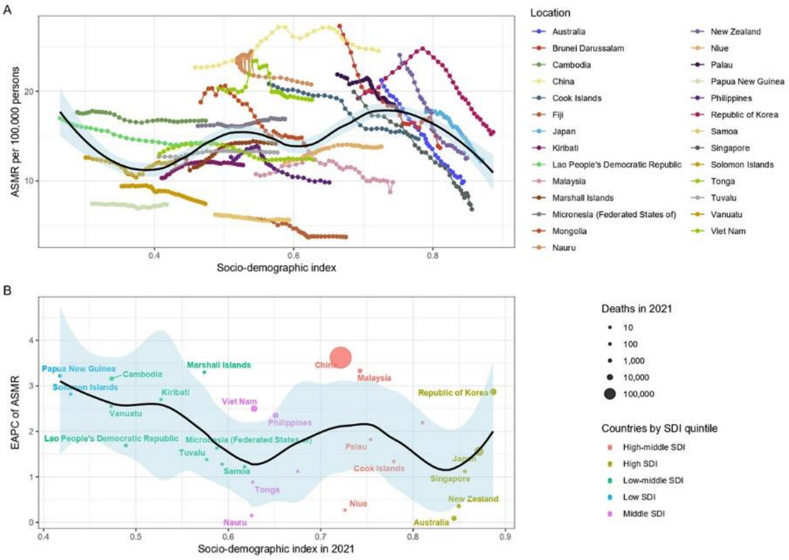

Supplementary Figure S3 The relationship between SDI levels and the age-standardized death rate for smoking-related lung cancer in the Western Pacific Region from 1990 to 2021

SDI: sociodemographic index.

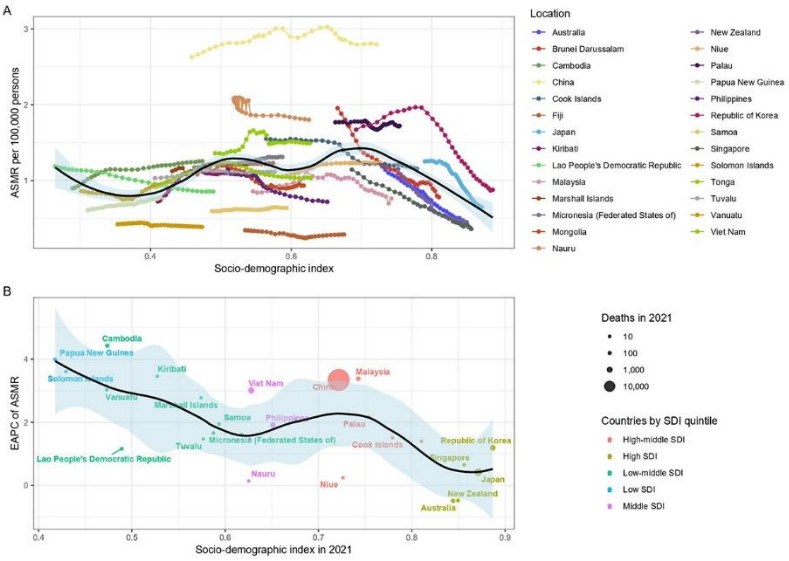

Supplementary Figure S4 The relationship between SDI levels and the age-standardized death rate for secondhand smoke-related lung cancer in the Western Pacific Region from 1990 to 2021

SDI: sociodemographic index.

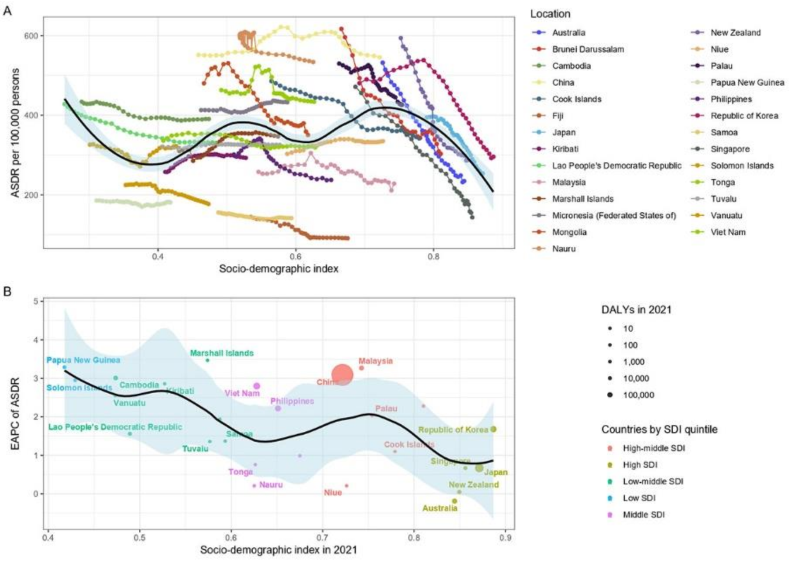

Supplementary Figure S5 The relationship between SDI levels and the age-standardized DALYs for smoking-related lung cancer in the Western Pacific Region from 1990 to 2021

SDI: sociodemographic index.

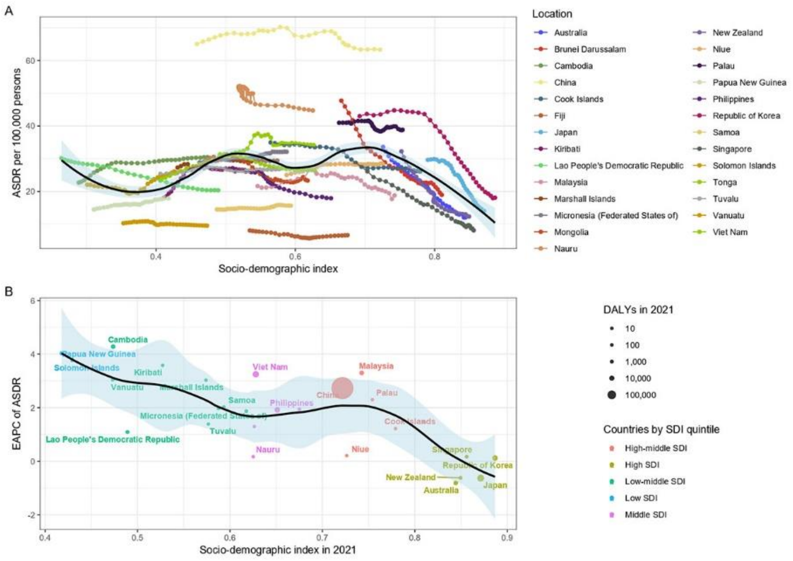

Supplementary Figure S6 The relationship between SDI levels and the age-standardized DALYs for secondhand smoke-related lung cancer in the Western Pacific Region from 1990 to 2021

SDI: sociodemographic index.

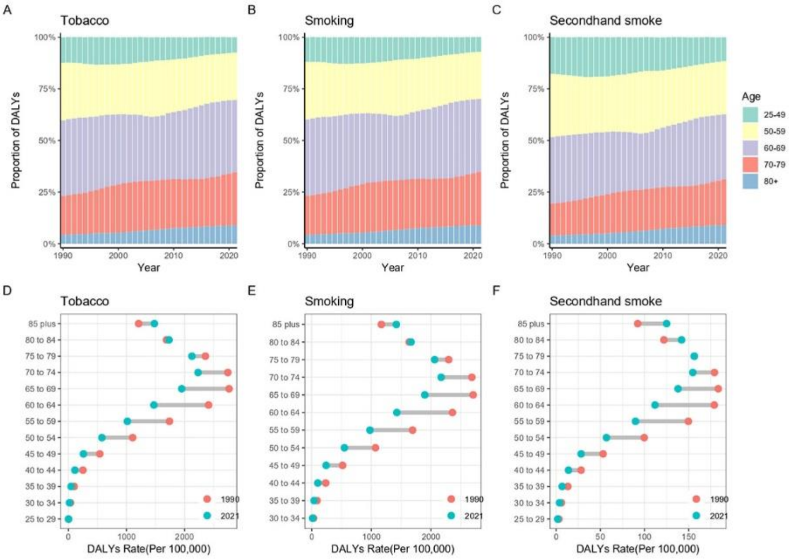

Supplementary Figure S7 The temporal change of the DALYs rate attributed to tobacco-related lung cancer across age groups in the Western Pacific Region from 1990 to 2021

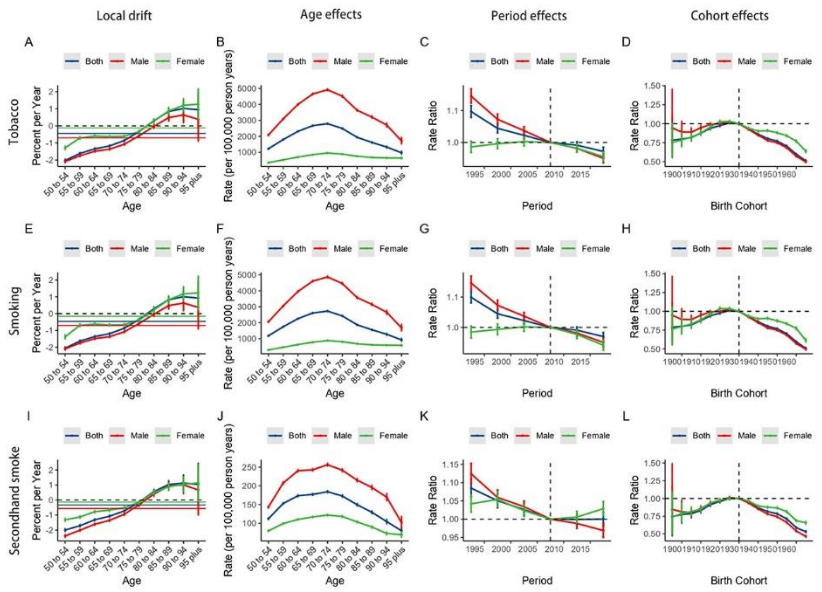

Supplementary Figure S8 The local drifts, age effects, period effects, and cohort effects of tobacco-related lung cancer related DALYs rate in the Western Pacific Region from 1990 to 2021.

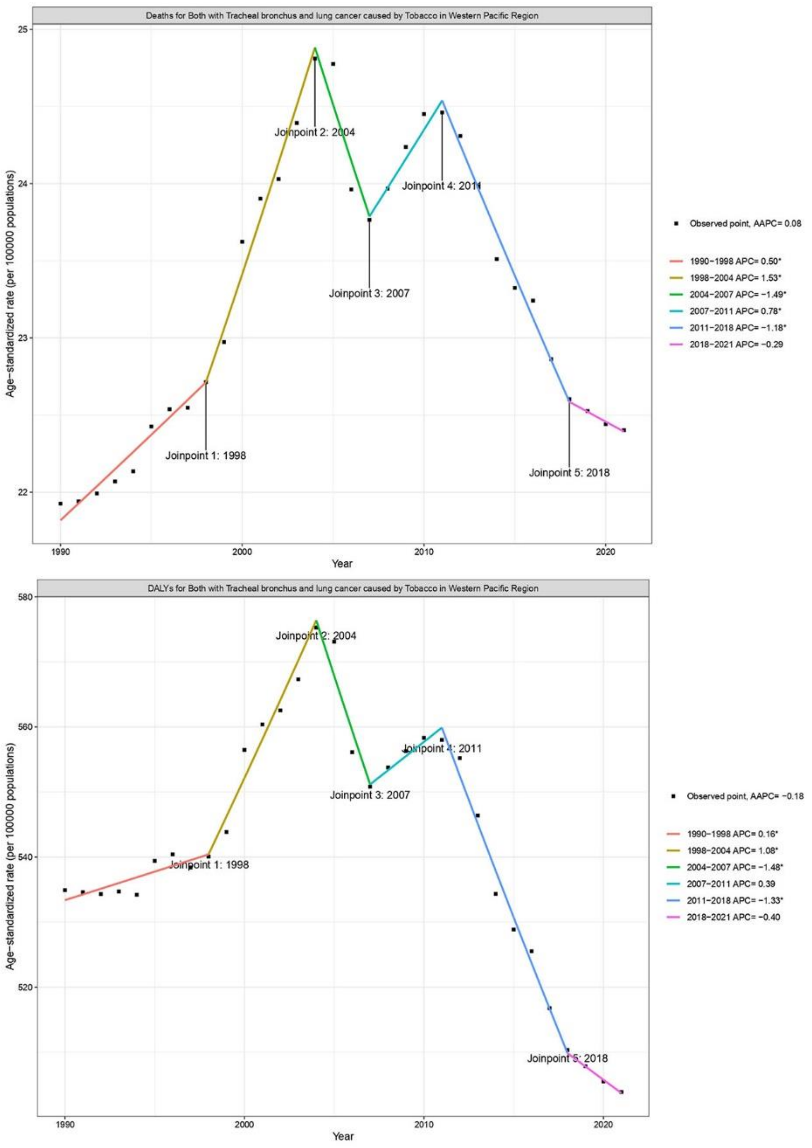

Supplementary Figure S9 The joinpoint regression analysis of the tobacco-related lung cancer burden in the Western Pacific Region from 1990 to 2021.

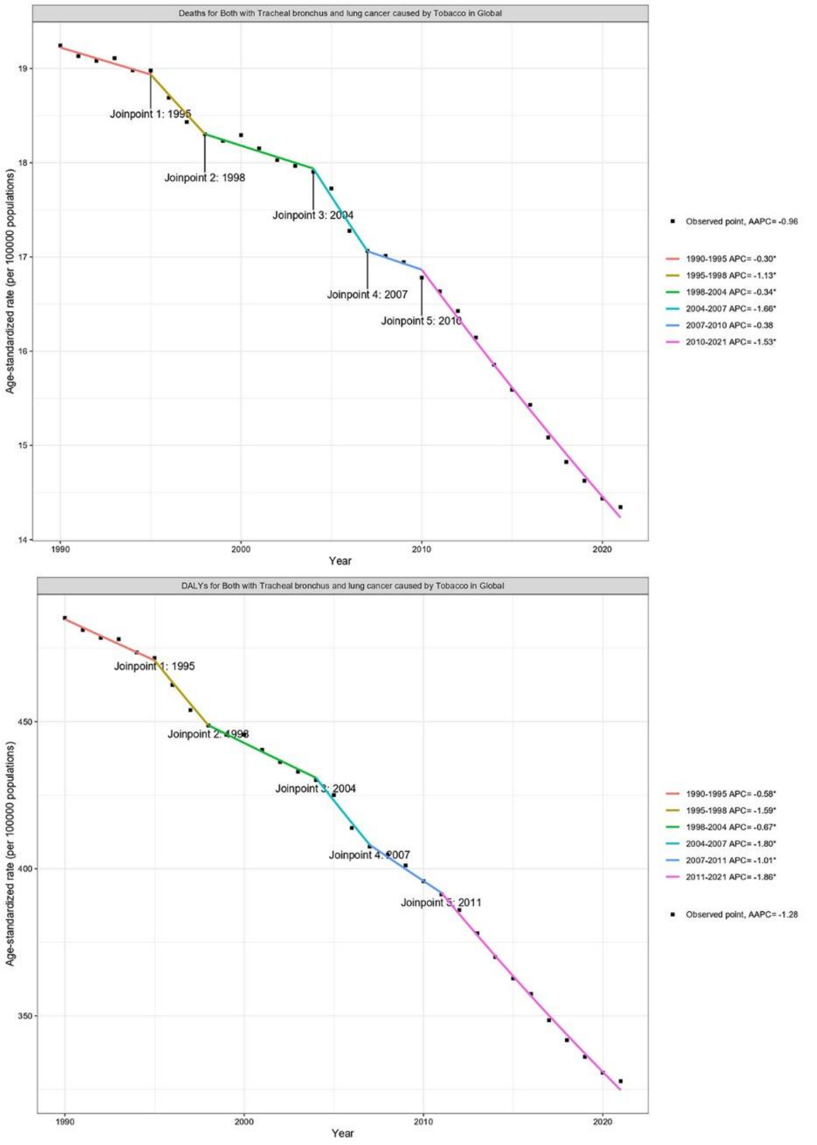

Supplementary Figure S10 The joinpoint regression analysis of the tobacco-related lung cancer burden in Global from 1990 to 2021.

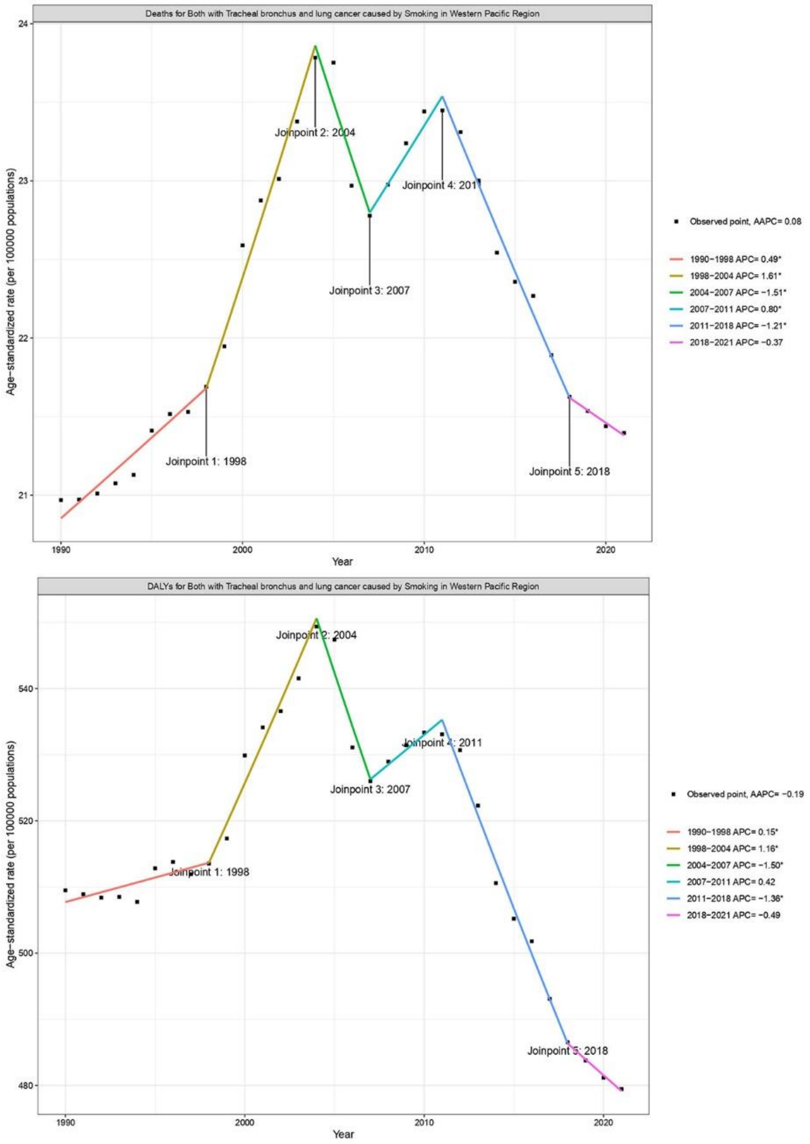

Supplementary Figure S11 The joinpoint regression analysis of the smoking-related lung cancer burden in the Western Pacific Region from 1990 to 2021.

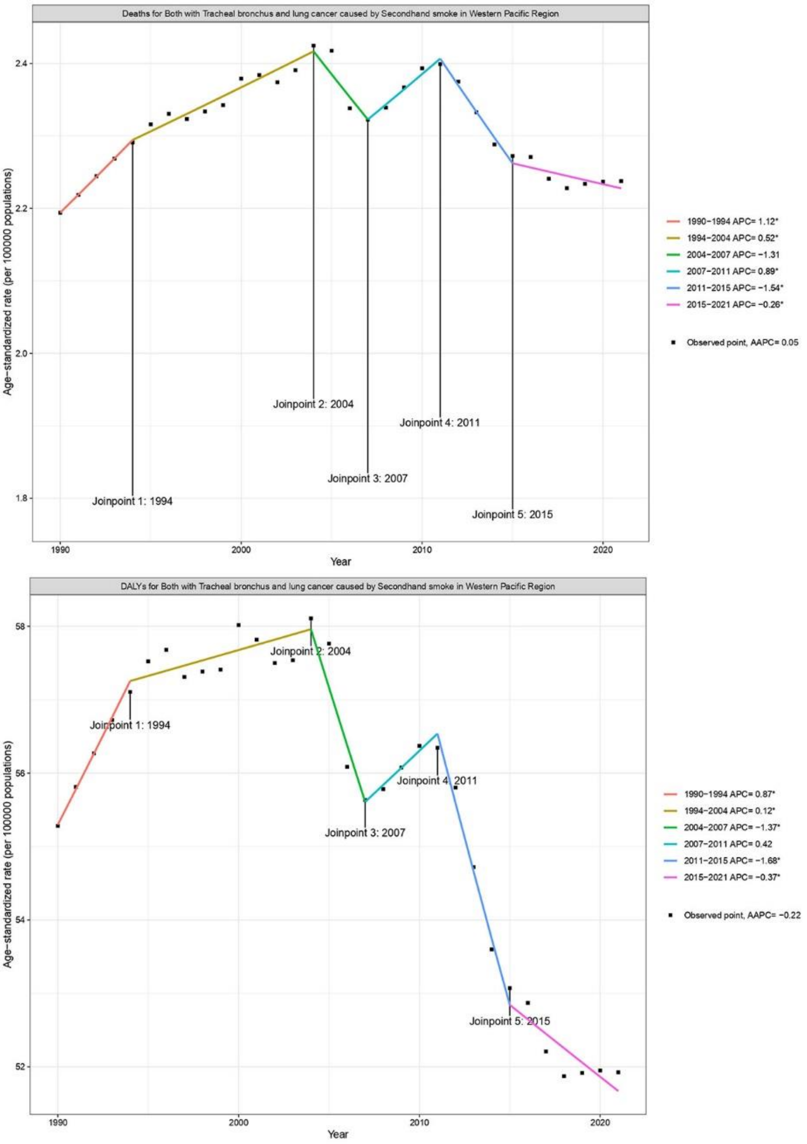

Supplementary Figure S12 The joinpoint regression analysis of the secondhand smoke-related lung cancer burden in the Western Pacific Region from 1990 to 2021.

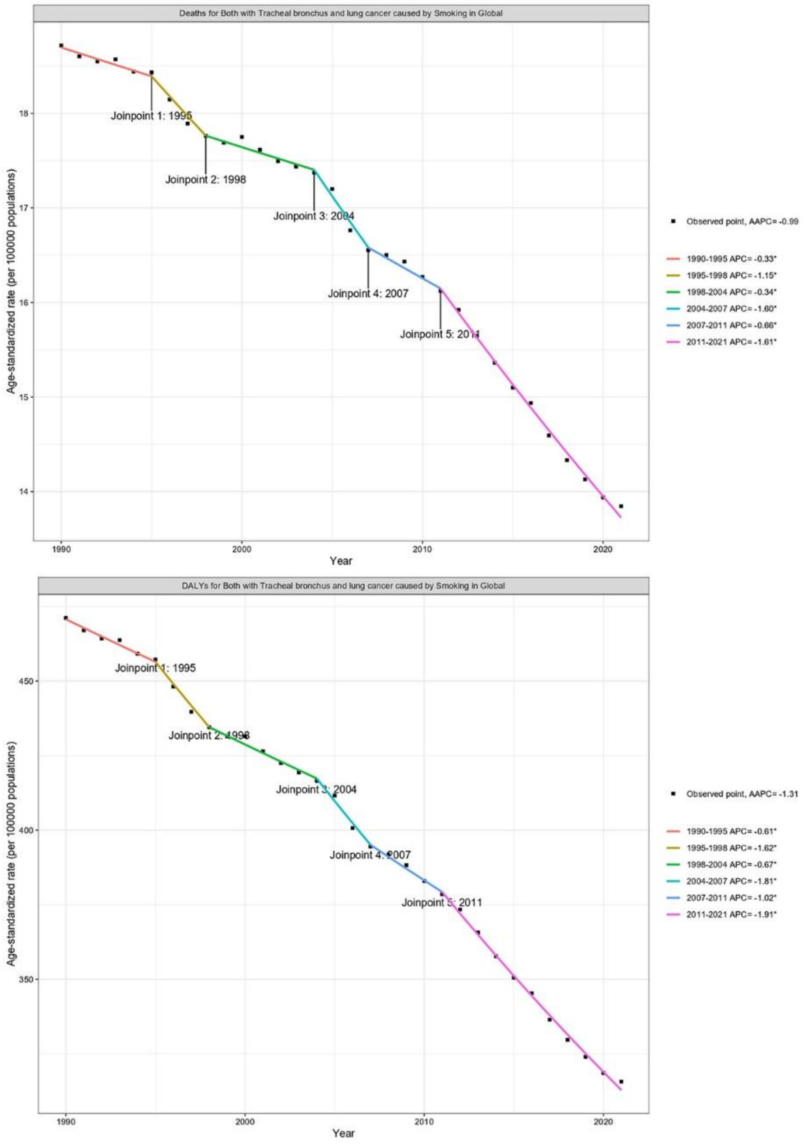

Supplementary Figure S13 The joinpoint regression analysis of the smoking-related lung cancer burden in Global from 1990 to 2021.

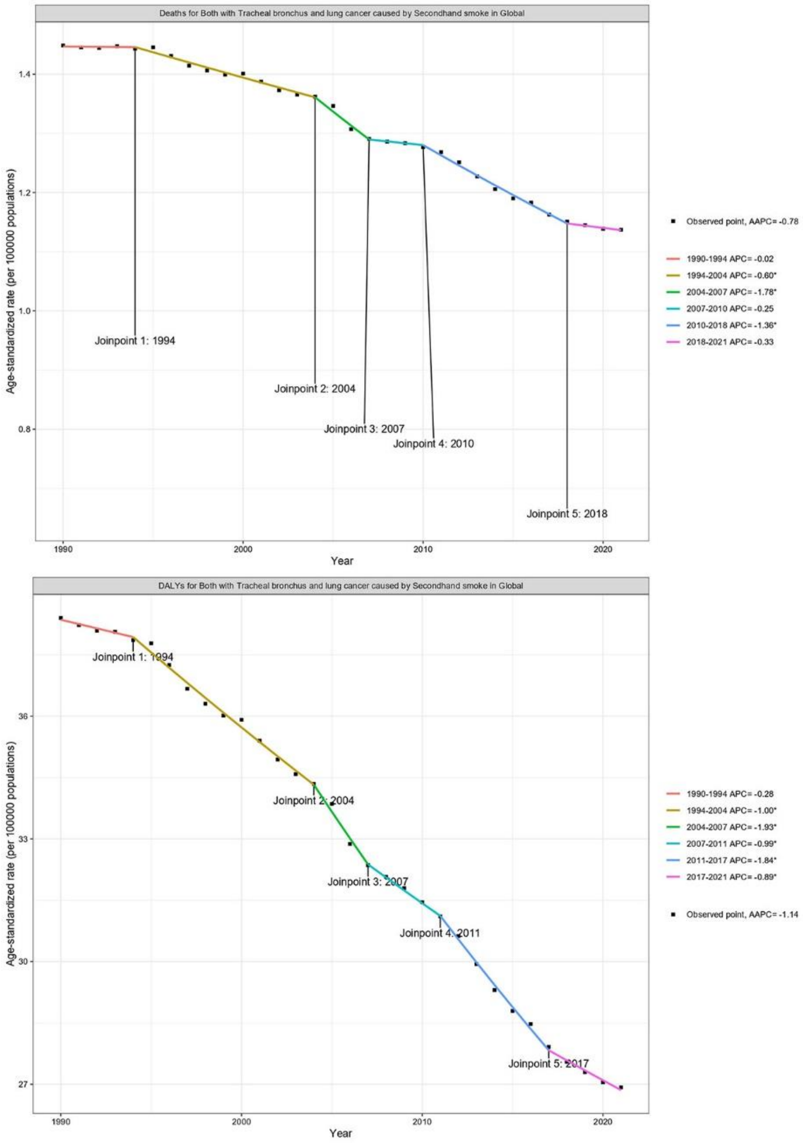

Supplementary Figure S14 The joinpoint regression analysis of the secondhand smoke-related lung cancer burden in Global from 1990 to 2021.

© 2025 Wang R. et al.
